# Effect of hospital volume on outcomes of total hip arthroplasty: a systematic review and meta-analysis

**DOI:** 10.1186/s13018-019-1531-0

**Published:** 2019-12-27

**Authors:** Syed Hamza Mufarrih, Muhammad Owais Abdul Ghani, Russell Seth Martins, Nada Qaisar Qureshi, Sayyeda Aleena Mufarrih, Azeem Tariq Malik, Shahryar Noordin

**Affiliations:** 1 Department of Biological and Biomedical Sciences, Aga Khan University, Karachi, Pakistan; 2 Department of Pediatric Surgery, Vanderbilt University Medical Center, Nashville, Tennessee USA; 3 Medical College, Aga Khan University, Karachi, Pakistan; 4 Department of Medicine, Aga Khan University, Karachi, Pakistan; 5 Medical College, Khyber Girls Medical College, Peshawar, Pakistan; 6 Department of Orthopedics, Ohio State University, Columbus, Ohio USA; 7 Department of Orthopedic Surgery, Aga Khan University, Karachi, Pakistan

**Keywords:** Total hip arthroplasty, Hospital volume, THA, Low-volume hospitals vs. high-volume hospitals, THA outcomes, Total hip replacement

## Abstract

**Background:**

A shift in the healthcare system towards the centralization of common yet costly surgeries, such as total hip arthroplasty (THA), to high-volume centers of excellence, is an attempt to control the economic burden while simultaneously enhancing patient outcomes. The “volume-outcome” relationship suggests that hospitals performing more treatment of a given type exhibit better outcomes than hospitals performing fewer. This theory has surfaced as an important factor in determining patient outcomes following THA. We performed a systematic review with meta-analyses to review the available evidence on the impact of hospital volume on outcomes of THA.

**Materials and methods:**

We conducted a review of PubMed (MEDLINE), OVID MEDLINE, Google Scholar, and Cochrane library of studies reporting the impact of hospital volume on THA. The studies were evaluated as per the inclusion and exclusion criteria. A total of 44 studies were included in the review. We accessed pooled data using random-effect meta-analysis.

**Results:**

Results of the meta-analyses show that low-volume hospitals were associated with a higher rate of surgical site infections (1.25 [1.01, 1.55]), longer length of stay (RR, 0.83[0.48–1.18]), increased cost of surgery (3.44, [2.57, 4.30]), 90-day complications (RR, 1.80[1.50–2.17]) and 30-day (RR, 2.33[1.27–4.28]), 90-day (RR, 1.26[1.05–1.51]), and 1-year mortality rates (RR, 2.26[1.32–3.88]) when compared to high-volume hospitals following THA. Except for two prospective studies, all were retrospective observational studies.

**Conclusions:**

These findings demonstrate superior outcomes following THA in high-volume hospitals. Together with the reduced cost of the surgical procedure, fewer complications may contribute to saving considerable opportunity costs annually. However, a need to define objective volume-thresholds with stronger evidence would be required.

**Trial registration:**

PROSPERO CRD42019123776.

## Background

Total hip arthroplasty (THA), a remarkably successful, safe, and cost-effective treatment for pain and joint dysfunction resulting from end-stage arthritis [1–4], is performed annually for approximately one million patients worldwide, with over 300,000 patients in the USA [5]. These numbers are expected to rise 174% by 2030, primarily driven by aging populations and an increase in life expectancy [5]. Although arthroplasty has shown promising results in reducing pain severity and improving the joint function of the patients, there is still room for improvement in terms of shortening length of hospital stay (LOS) and decreasing risk of postoperative dislocation, peri-prosthetic fracture, and infection to effectively lower the overall cost of hip arthroplasty and revision rates.

Since the inception of modern THA in 1960 [6], various studies have identified several factors that may affect the outcomes of the surgery. These include *patient-related* factors such as age [7, 8], gender [9, 10], elevated body mass index [11], number of comorbid conditions [12, 13], American Society of Anesthesiologist (ASA) grade [14], neuropsychiatric disorders [15, 16], and *technical-factors* such as surgical complexity, implant type, head size, and bearing surfaces [17–20].

Recently, the concept of *hospital factors* has surfaced. It has been shown that hospitals performing more treatments of a given type exhibit better outcomes than hospitals performing fewer. This is called the “volume-outcome” relationship and several studies have observed this effect in total hip arthroplasty, where the outcomes of hip arthroplasty in hospitals which perform a higher number of hip arthroplasty procedures annually are better than hospitals which perform a fewer number [21–23]. As the current healthcare system endeavors to implement value, centralization of common yet costly surgeries, such as THAs, to high-volume centers of excellence may be an effective way to control the economic burden. While several studies have investigated the hospital volume relationship, no systematic review or meta-analysis has been conducted to pool the results. In our study, we combine data from all published studies to study the differences in outcomes of hip arthroplasty in high-volume and low-volume hospitals.

## Methods

The review follows the PRISMA guidelines [24].

### Search strategy

A review of PubMed (MEDLINE), OVID MEDLINE, Google Scholar, and Cochrane library review was conducted for studies reporting the effect of hospital volume on outcomes of total hip arthroplasty (THA), since 1980 to March 2019. In order to yield maximum results, the keywords used were (“hospital volume” OR (“hospital” AND “volume”) AND (“total hip arthroplasty” OR “THA” OR “total hip replacement” OR “hip replacement”). Duplicates were removed and titles of all studies were screened as per the eligibility criteria. Any ambiguity was resolved through screening the abstract. The full text of articles that met the inclusion criteria was reviewed. The references of the selected studies were screened for potentially relevant studies.

### Selection criteria

Studies reporting the effect of hospital volume on total hip arthroplasty, published in English, with available full texts, were selected. The inclusion criteria and the exclusion criteria have been summarized in Table 1. Two authors (SHM and ATM) independently screened all abstracts from the initial search to assess eligibility for inclusion.

**Table 1:** Eligibility Criteria for studies included in the review

Inclusion criteria	Exclusion criteria
1) Studies that compared the outcomes of low-volume hospitals (LVH) and high-volume hospitals (HVH) for hip arthroplasty. 2) Articles in which the study population was undergoing primary or revision THA.* 3) Reported outcomes included perioperative morbidity/complication, in-hospital mortality, postoperative mortality within 1 year, readmission, length of stay (LOS), and cost of surgery.	1) Less than 25 cases 2) Greater than 10% patients lost to follow-up 3) Measured outcomes not reporting significance of results 4) Studies not available in English

*Studies reporting relationships between hospital volume and hip arthroplasty following trauma/malignancy were excluded.

### Data extraction

Data extraction was done by two authors (MAOG and RSM) independently using Excel 2011 software. Data extraction variables were pretested using five papers. The extracted parameters included author name, study design, study duration, number of hips included, reported outcomes, mean age of study population, adjustment for covariates, and percentage of patients lost to follow-up. In addition to this, the cut-off for categorizing hospital volume as high or low, reported complications, the OR/RR or HR values along with their confidence interval and *p* value were also extracted.

The number of patients in low-volume hospital (LVH) and high-volume hospital (HVH) groups and complications (e.g., mortality, surgical site infection) were extracted for the meta-analysis from each study. In case, raw data in terms of crude numbers was not reported, efforts were made to contact the author via email to request them to provide us with the data necessary for the inclusion of their study in the pooled analysis.

### Synthesis of results

The meta-analysis was performed using RevMan Version 5.3 (The Cochrane Collaboration, Copenhagen, Denmark) for calculating pooled summaries and generating forest plots. Meta-analysis was only possible if the retrieval of sufficient data from the study or through contact with the author had been successful.

There was considerable heterogeneity among the studies in the cutoffs for categorizing hospital volume as low or high. To account for this, and other variations including the fact that studies were performed in different regions of the world, with differences in age groups and technical surgical protocols, we decided to use the Mantel-Haenszel random-effect model to report the risk-ratio and heterogeneity (*I*^2^) in our analysis.

The random effect model assumes that the effect size is obtained from a population of effect sizes. Therefore, the effect size is derived from the sampling of an effect size at random, in addition to measurement error (the inverse function of the sample size). Because the random-effects model considers the two sources of error in effect size, they are able to yield a larger error term and less statistical power than fixed-effect models. However, one could benefit from random effect models over fixed-effect models because of its ability to generalize the result to a broader universe of studies. These models specifically account for the heterogeneity of studies through a statistical parameter representing the inter-study variation. For the parameters recorded in our review, the random-effect model was preferred for the pooling of the data in the meta-analysis.

For pooling means, we used the standard mean difference (SMD) instead of mean difference, so that we could standardize the results of all studies to a uniform scale. This was necessary as the mean values varied widely from region to region (For example, the mean length of stay in studies from Japan ranged between 25–60 days postoperatively while the mean length of stay in studies in the USA ranged between 4–10 days).

The use of standard mean difference expresses the size of the intervention effect in each study relative to the variability observed in the study, rather than comparing it with other studies. Therefore, the net effect determined is the pooled summary of the standard mean difference among each individual study, rather than a comparison with the means of other studies. This was used to compare the difference in length of stay and cost of surgery between HVH and LVH. The length of stay was reported by many studies in different parts of the world, and the protocols they use for their surgeries vary vastly. Similarly, the cost of the surgeries was reported using different currencies at different times. Such a model is useful to pool studies with such vast heterogeneity.

We decided to pool surgical site infections as per the CDC definition of surgical site infection, 1999 [25].

### Quality appraisal of studies

We used the Grading of Recommendations Assessment, Development and Evaluation (GRADE) system to evaluate the quality of studies in our review [26].

## Results

This work has been reported in accordance with the PRISMA (Preferred Reporting Items for Systematic Reviews and Meta-Analyses) [27] and AMSTAR (Assessing the methodological quality of systematic reviews) Guidelines [28].

### Study selection

**Fig. 1:**
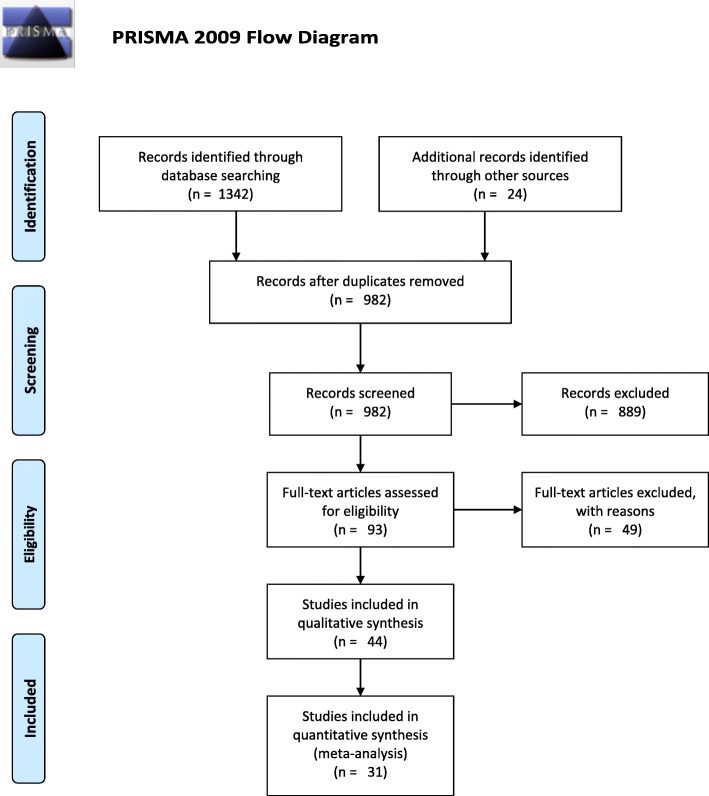
PRISMA flow diagram for study selection

A preliminary total of 1342 studies were identified from Google Scholar, PubMed, and Cochrane library. The removal of duplicates yielded 982 studies. Through screening of titles and abstracts, 93 studies which fulfilled the inclusion criteria were extracted. Following full-length reviews, 49 studies were excluded based on the exclusion criteria. Forty-four studies were included in the qualitative review, and only 31 were included in the meta-analysis. The process of study selection has been summarized in the PRISMA flow diagram (Fig. 1: PRISMA flow diagram for study selection).

### Study characteristics

A total of 44 studies were included in the review. Forty-two studies were retrospective studies where longitudinal data was collected over a certain period of time from pre-existing databases, while 2 were prospective. Six studies had included both primary THA and revision THA [23, 29–33]. The remaining 38 studies were focused solely on primary THA. Data from revision surgeries is described in Additional file 4. Only data from primary THA was used to pool results in our analyses. Twenty-four of the 44 studies were conducted in the USA while the remaining were contributed by 11 unique countries (details in Additional file 1). The average age of the patient population was 67.7 years (reported by 28 studies). Overall, 43.3% of the patients were male and 56.7% of the patients were female (reported by 34 studies). Details of the study characteristics are summarized in Additional file 1. A total of 38 studies had adjusted for covariates (details in Additional file 2). Patient comorbid conditions including diabetes mellitus, obesity, dyslipidemia, chronic kidney disease, heart disease, hypothyroidism, chronic obstructive pulmonary disease, peripheral vessel disease, and depression were only reported by 13 studies (details in Additional file 5).

### Outcomes and findings

These studies include data from 1988 to 2011. Detailed results of data extraction on reported outcomes are presented in Additional file 3.

#### Surgical-site infections

**Fig. 2:**
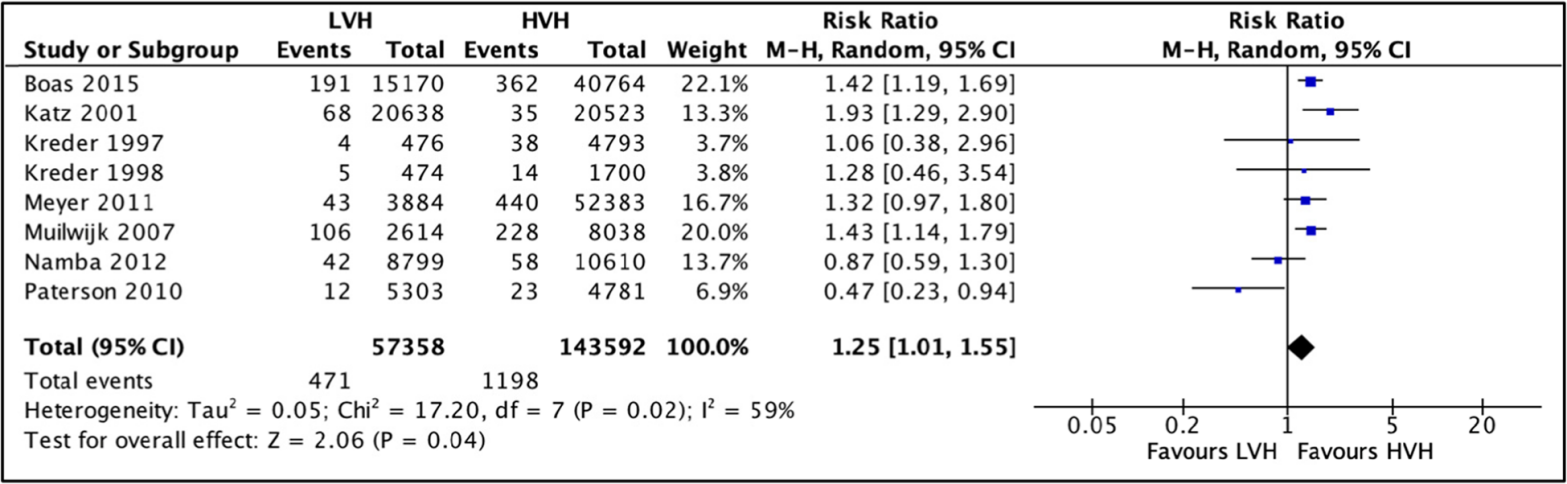
Comparison of surgical site infections (1-year post-operatively) between low-volume and high-volume hospitals

A total of 8 studies [31, 34–40] totaling 200,950 hip arthroplasties were pooled to compare the rates of surgical-site infections SSI 1-year postoperatively between LVH and HVH. We observed that surgical site infections were more frequently observed in LVH with a risk ratio (RR) of 1.25 (CI [1.01, 1.55]; *I*^2^ = 59%, *p* value = 0.04) (Fig 2 comparison of surgical site infections (1-year postoperatively) between low-volume and high-volume hospitals**)**.

Four studies, which could not be added to the pooled-analysis, also compared postoperative incidence of SSIs. Two of the studies (Kaneko, et al. [41] and Soohoo et al. [42]) reported a significantly higher rate of SSIs following THA at low-volume hospitals while two studies (Huang et al. [43]. and Makela et al. [44]) reported no significant difference between low-volume and high-volume hospitals.

#### Cost of surgery

**Fig. 3:**
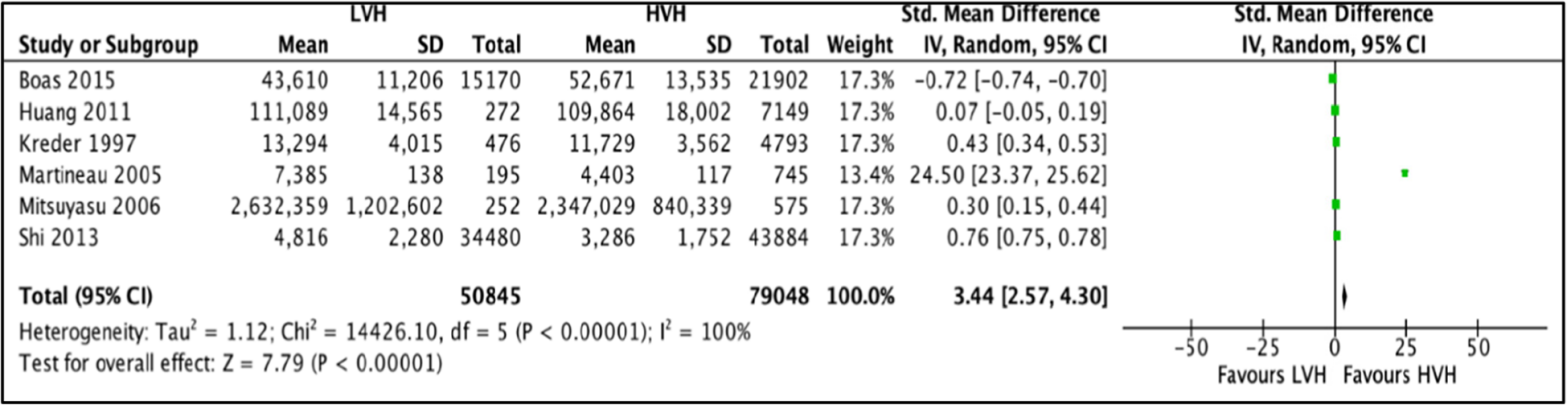
Comparison of cost of surgery between low-volume and high-volume hospitals

Six studies [34, 36, 43, 45–47] totaling 129,893 hip arthroplasties were pooled to compare the cost of Primary THA in LVH vs. HVH. Based on the results of the random meta-analysis model, we found that the cost of surgery is significantly higher in LVH with SMD of 3.44 (CI [2.57, 4.30]; *I*^2^ = 100%, *p* value < 0.00001) (Fig. 3: comparison of cost of surgery between low-volume and high-volume hospitals**).**

Three studies reporting differences in the cost of surgery could not be included in the meta-analysis. Courtney et al. [48] reported that THA procedures performed at HVH had significantly lower total mean hospital-specific charges. Frisch et al. [49] and Lavernia et al. [50] found no significant difference between mean hospital charges for THA performed at LVH and HVH.

#### Length of postoperative hospital stay

**Fig. 4:**
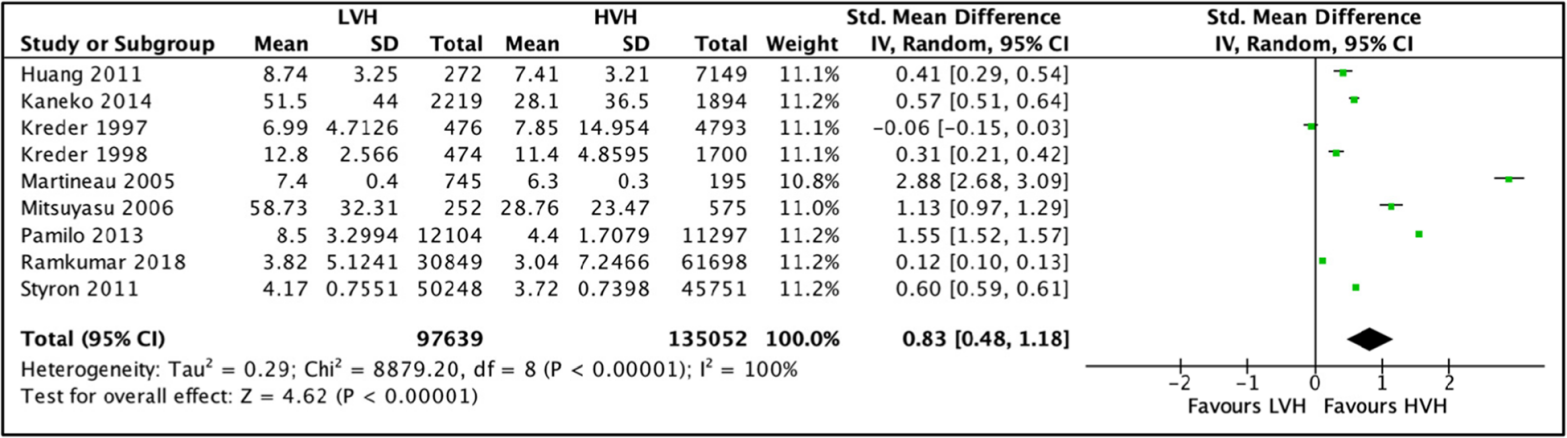
Comparison of length of stay between low-volume and high-volume hospitals

A total of 9 studies [36, 37, 41, 43, 45, 46, 51–53] totaling 232,691 hip arthroplasties were pooled to compare the length of postoperative stay in LVH vs. HVH. Based on the results of the random meta-analyses model, we found that postoperative stay was significantly longer in LVH with a SMD of 0.83 (CI [0.48, 1.18] *I*^2^ = 100%, *p* value = 0.00001) (Fig. 4: comparison of length of stay between low-volume and high-volume hospitals.**)**.

Three studies reporting length of stay in low-volume and high-volume hospitals could not be included in the meta-analysis. Doro, et al. [23], Makela et al. [44], and Judge et al. [54] reported increased LOS in LVH as compared to VHVH.

#### Complications during index hospitalization

**Fig. 5:**
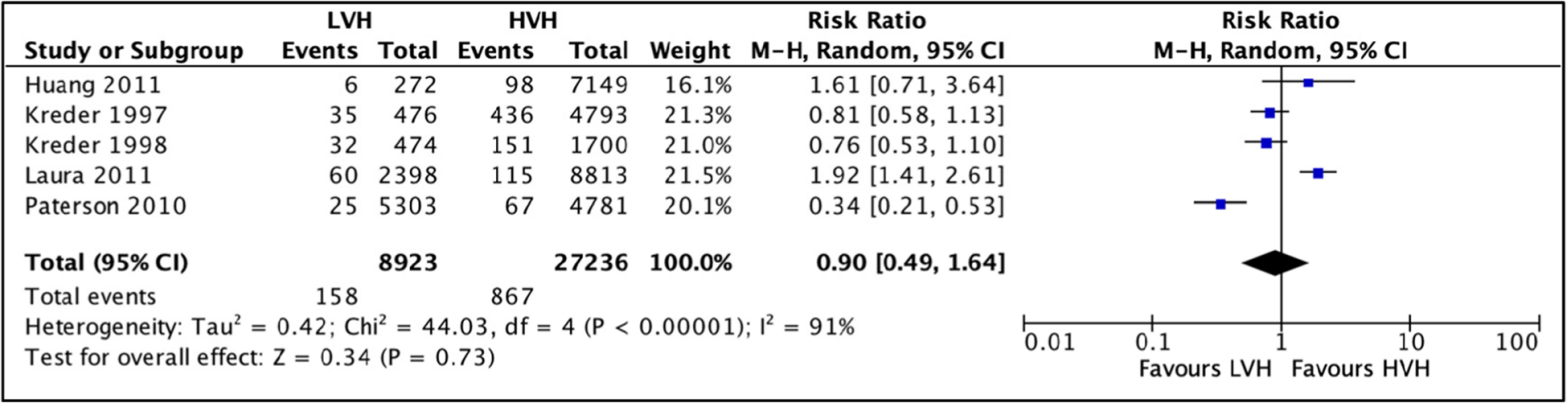
Comparison of complications during index hospitalization between low-volume and high-volume hospitals

A total of 5 studies [36, 37, 40, 43, 55] totaling 36,159 hip arthroplasties were pooled to compare the complications during index hospitalization between LVH and HVH. Based on the results of the random meta-analysis model, we found that there is no significant difference in rates of index hospitalization complications between LVH and HVH, with RR = 0.90 (CI [0.49, 1.64] *I*^2^ = 91%, *p* value = 0.73) (Fig. 5: comparison of complications during index hospitalization between low-volume and high-volume hospitals.).

#### Complications within 90 days postoperatively

**Fig. 6:**
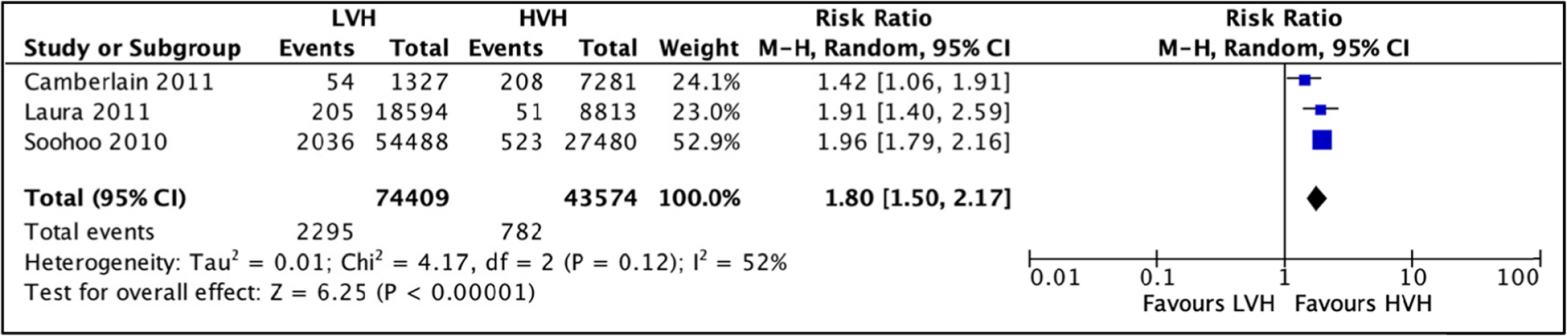
Comparison of complications 90 days postoperatively between low-volume and high-volume hospitals

A total of 3 studies [42, 55, 56] totaling 74,409 hip arthroplasties were pooled to compare the rates of complications 90 days postoperatively in LVH with HVH. Based on the results of the random meta-analyses model, we found that there is a significantly higher chance of 90-day complications in LVH as compared to HVH (RR = 1.80 (CI [1.50, 2.17] *I*^2^ = 52%, *p* value < 0.00001) (Fig. 6: comparison of complications 90 days postoperatively between low-volume and high-volume hospitals**).**

Although not included in the meta-analysis, Solomon et al. and Katz (2001) et al. also report a higher incidence of postoperative complications in LVH compared to HVH.

#### Revision hip arthroplasty at 1-year postoperative

**Fig. 7:**
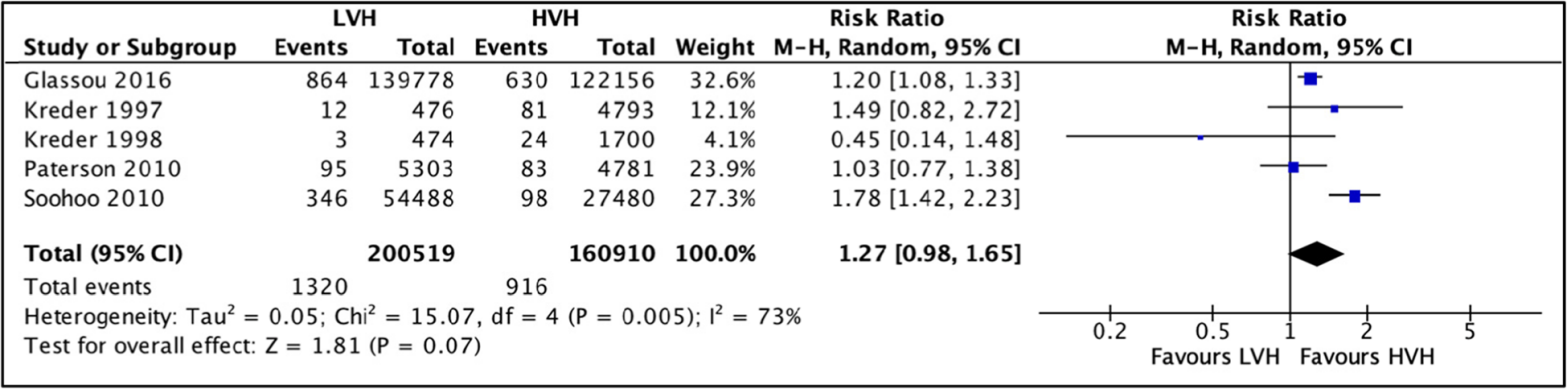
Comparison of revision hip arthroplasty 1 year postoperatively between low-volume and high-volume hospitals

A total of 5 studies [36, 37, 40, 42, 57] totaling 361,440 hip arthroplasties were pooled to compare the rates of revision THA between LVH and HVH. Based on the pooled analysis, no significant difference was observed between rate of revisions for surgeries performed in LVH and HVH 1 year postoperatively; RR = 1.27 (CI [0.98, 1.65] *I*^2^ = 73%, *p* value = 0.07) (Fig. 7: comparison of revision hip arthroplasty 1 year postoperatively between low-volume and high-volume hospitals**).**

#### Revision hip arthroplasty at 3 years postoperative

**Fig. 8:**
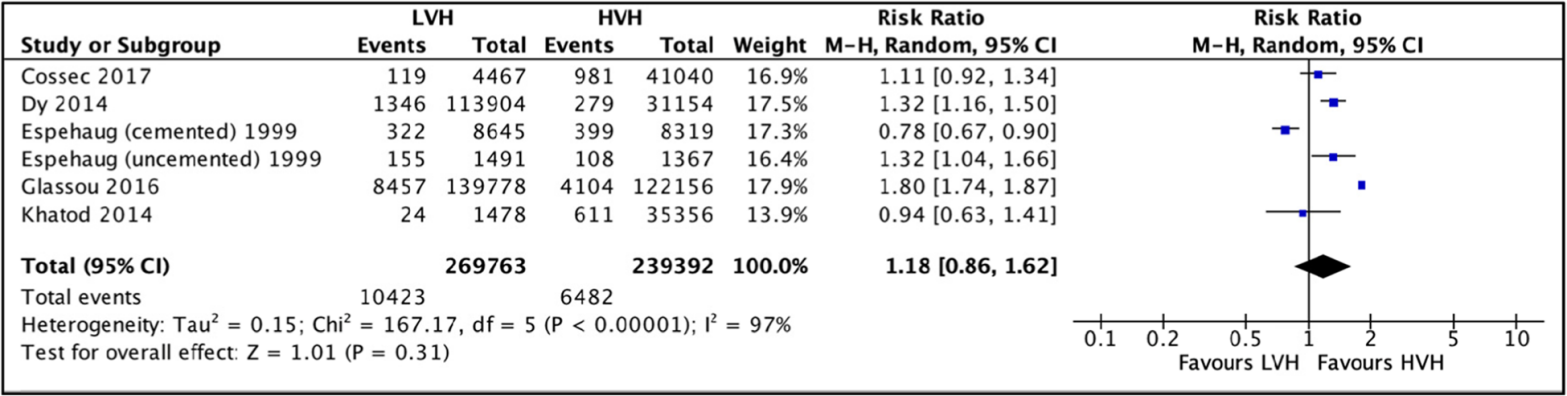
Comparison of revision hip arthroplasty 3-year postoperative between low-volume and high-volume hospitals

Five studies [22, 57–60] totaling 509,155 hip arthroplasties were pooled. No significant difference was observed between rate of revisions for surgeries performed in LVH and HVH; RR = 1.18 (CI [0.86, 1.62] *I*^2^ = 97%, *p* value = 0.31) (Fig. 8: comparison of revision hip arthroplasty 3 years postoperative between low-volume and high-volume hospitals).In addition to this, Pamilo et al. [51], Makela et al. [44], and Manley et al. [61] also reported that there was no significant association between revision rates and hospital volume. In contrast, Judge et al. [54] reported a higher hazard ratio of revision arthroplasty 5 years postoperatively for HVH vs. LVH.

#### 30-day mortality

**Fig. 9:**
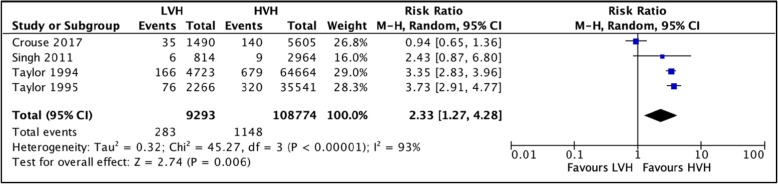
Comparison of 30-day mortality between low-volume and high-volume hospitals (study by Taylor et al. reported findings from 1994 to 1995 separately)

Three studies [21, 33, 62] totaling 140,656 hip arthroplasties were pooled to compare the mortality rates within 30 days postoperatively between LVH and HVH. Based on the results of the random meta-analysis model, we found a significantly higher mortality rate in LVH, RR = 2.33 (CI [1.27, 4.28] *I*^2^ = 93%, *p* value = 0.006) (Fig. 9: comparison of 30-day mortality between low-volume and high-volume hospitals (study by Taylor et al. reported findings from 1994 and 1995 separately**).**

#### 90-day mortality

**Fig. 10:**
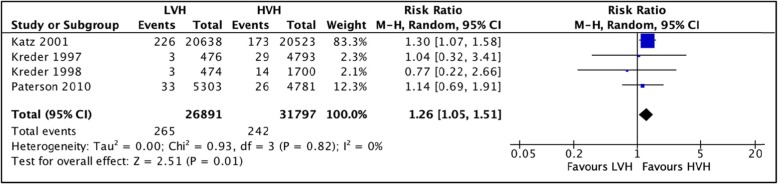
Comparison of 90-day mortality between low-volume and high-volume hospitals.

A total of 4 studies [35–37, 40] totaling 58,688 hip arthroplasties were pooled to compare mortality rates within 90 days postoperatively between LVH and HVH. Based on the results of the random meta-analysis model, we found a significantly higher mortality rate in LVH, RR = 1.26 (CI [1.05, 1.51] *I*^2^ = 0%, *p* value = 0.01) (Fig. 10: comparison of 90-day mortality between low-volume and high-volume hospitals.**).**

#### 1-year mortality

**Fig. 11:**
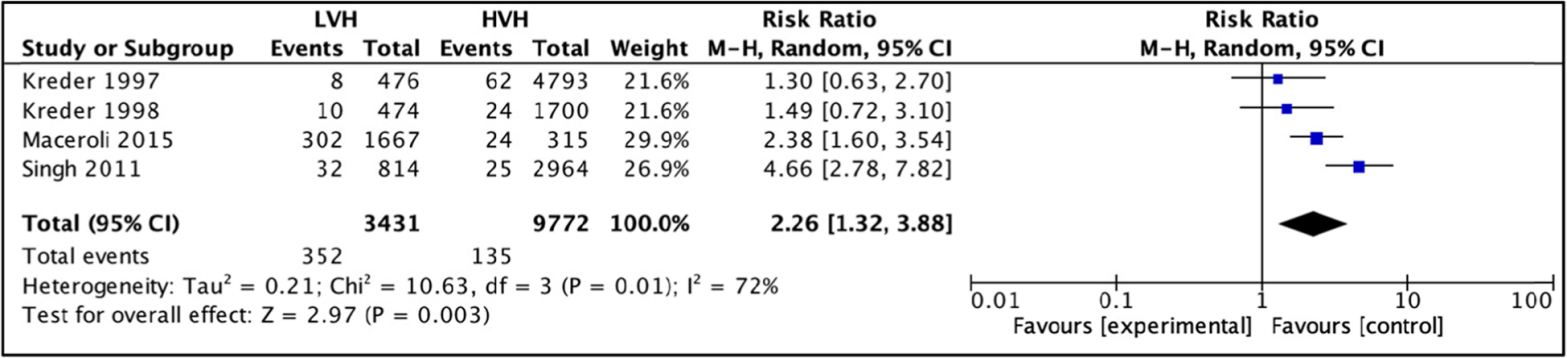
Comparison of 1-year mortality between low-volume and high-volume hospitals

Four studies, [36, 37, 62, 63] totaling 13,203 hip arthroplasties were pooled to compare mortality rates within 1 year postoperatively between LVH and HVH. Based on the results of the random meta-analysis model, we see that there is a significantly higher mortality rate in LVH, RR = 2.26 (CI [1.32, 3.88] *I*^2^ =72%, *p* value = 0.003) (Fig. 11: comparison of 1-year mortality between low-volume and high-volume hospitals.**).**

A few studies reporting postoperative mortality as an outcome could not be included in the meta-analysis. Two studies (Lavernia et al. [50] and Laura et al. [55]) stated no statistically significant difference in mortality rates between LVH and HVH. However 7 studies (Hughes et al. [64], Solomon et al. [65], Sharkey et al. [66], Riley et al. [67], Judge et al. [54], Chien et al. [68], and Doro et al. [23]) reported a significant inverse relation between hospital volume and mortality rate.

#### Postoperative thromboembolic events

**Fig. 12:**
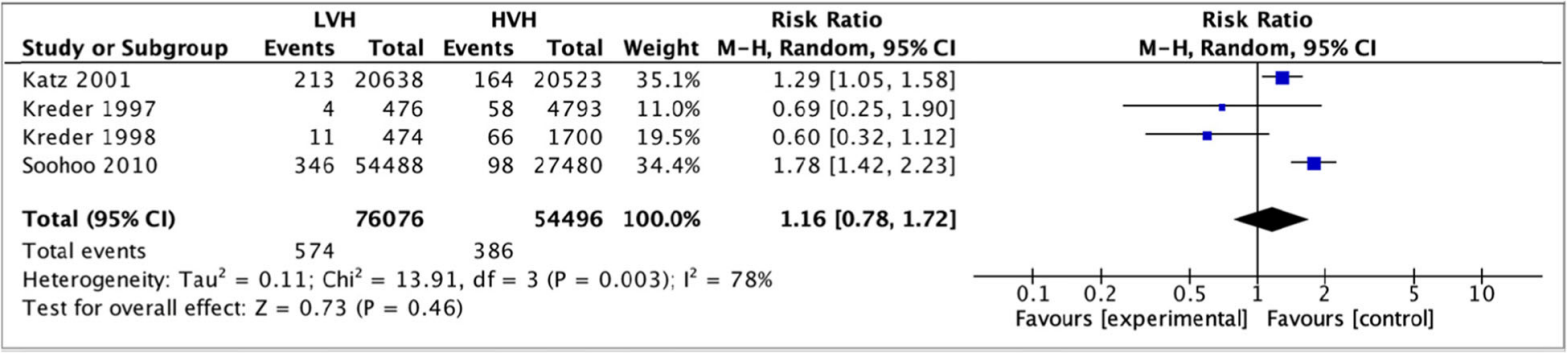
Comparison of postoperative deep venous thrombosis between low-volume and high-volume hospitals

Five studies, [35–37, 42] totaling 130,572 hip arthroplasties were pooled to compare the rates post-operative deep venous thrombosis between LVH and HVH. Based on the results of the random meta-analysis model, we found that there was no significant difference in the risk of thromboembolic events 90 days postop; (RR = 1.16, (CI [0.78, 1.72] *I*^2^ = 78%, *p* value = 0.46) (Fig. 12: comparison of postoperative deep venous thrombosis between low-volume and high-volume hospitals.**).**

### Quality appraisal

All the studies included in this review are observational studies, with 42 retrospective and 2 prospective, which are considered low-grade studies as per the GRADE system guidelines.

## Discussion

Few systematic reviews have been conducted in the realm of orthopedic surgery to study the volume-outcome relationship. While knee arthroplasty [69], shoulder arthroplasty [70], spine surgery [71], and hip fractures [72] have been extensively studied, evidence of the effect of hospital volume on total hip arthroplasty has not been reviewed systematically to our knowledge.

Our review was based on observational studies from several countries around the world with a predominant contribution from the USA. In our review, we found that THAs performed at LVH have a significantly higher risk of surgical site infections, cost of surgery, length of stay, 90-day complications, and mortality (30-days, 90 days, and 1 year) (Table 2).

**Table 2:** Summary of findings

Outcome	Studies	Hips	Risk ratio	Significance
Postoperative surgical site infection	8	200,950	1.25 CI [1.01, 1.55]	0.04
Cost of surgery	6	129,893	3.44 CI [2.57, 4.30]	< 0.00001
Postoperative length of hospital Stay	9	232,691	0.83 CI [0.48, 1.18]	< 0.00001
Complications during index hospitalization	5	36,159	0.90 CI [0.49, 1.64]	0.73
Complication within 90 days post-op	3	74,409	1.80 CI [1.50, 2.17]	< 0.00001
Revision arthroplasty within 1 year post-op	5	361,440	1.27 CI [0.98, 1.65]	0.07
Long-term revision arthroplasty	5	509,155	1.18 CI [0.86, 1.62]	0.31
30-day mortality	3	140,656	2.33 CI [1.27, 4.28]	0.006
90-day mortality	4	58,688	1.26 CI [1.05, 1.51]	0.01
1-year mortality	4	13,203	2.26 CI [1.32, 3.88]	0.003
Postoperative thromboembolic events	5	147,015	1.28 CI [0.92, 1.77]	0.15

*CI* confidence interval

*Risk ratio for low-volume hospitals in comparison to high-volume hospitals

We found a significantly higher risk of postoperative surgical site infections (SSIs) in LVH compared with HVH. The finding of SSI risk being higher in LVH as compared to HVH may be linked to other factors. Previous literature has reported the association of SSIs with a longer length of hospital stay and has linked a longer length of stay to low-volume hospitals [73, 74]. Another risk factor associated with SSIs is longer operative duration which has also been observed in low-volume hospitals in previous literature [31, 75, 76]. In addition, previous literature suggests that high-volume hospitals may enjoy superior infection prevention measures [77]. Our results, though specific to THA, are in agreement with findings of prior research showing higher risks of SSI in LVH [78, 79].

Our findings also show an inverse relationship between hospital volume and cost of surgery. This may be attributed to greater negotiating power of high-volume hospitals because of higher numbers, greater efficiency, and accelerated care pathways allowing expedited discharge processes and more prudent use of ancillary services at HVH resulting in significant cost savings for the healthcare system and the patient [80].

Length of stay may be a complex variable to dissect as it is a combined reflection of pre-operative, intra-operative, and postoperative care. Previous literature has reported associations among operative time, postoperative complications, and length of stay [81–83]. Though we did not find a significant difference between the postoperative complications in HVH and LVH during their hospital stay in our study, the key factors underlying this complex relationship are potentially related to the superior healthcare provision capacity of HVH including availability of special care facility, infrastructure, specialist medicine care, physiotherapy, pain control anesthesia teams, and other resources during all stages of care and health economy of the country the study was conducted in [35, 84]. This enables the hospitals to be better equipped to deal with problems before they escalate to serious complications [85, 86]. Although several intra-operative factors may also play a role in determining the length of stay between HVH and LVH, this has not been studied in detail.

Although our findings show no significant difference between complications during the hospital stay, complications at 90 days were reported to be significantly higher in LVH when compared to HVH. Soohoo [42] studied this extensively and concluded that patient and surgeon factors heavily influenced the risk of developing complications. Patient factors associated with higher 90-day complications included male gender, higher Charlson comorbidity score, comorbid conditions such as diabetes and rheumatoid arthritis [42]. While these may not be under the control of the hospital, a shorter learning curve [87] in the presence of better resources [85, 86, 88] may allow an increase in the capacity of HVH to be proactive in identifying and resolving issues before they can adversely influence outcomes. Although our results show a higher 90-day complication rate with low-volume hospitals, there is no significant difference in 1-year and 3-year revision rates between LVH and HVH. This may be due to possible loss to follow-up, visit to high-volume hospitals for revisions or mortality (as seen in our findings).

From a monetary perspective, not only are hip replacement surgeries at HVH cost-effective, they also have a greater value per dollar spent in the long run as they are associated with lower rates of complications, especially surgical site infections. SSIs result in significant losses with up to three-fold cost increase after orthopedic surgeries [89]. This is particularly alarming as the rates of surgical site infections in the USA are on the rise [5, 90–92] and reimbursements are being reduced or denied [93]. Lower rates of 90-day complications are not only better outcomes, but also saves the costs of readmission. Additionally, HVH may have room to negotiate costs with suppliers due to their large volumes. This translates into decreased costs which benefit the patients and the healthcare system.

In contrast, longer length of stay associated with LVH following THA procedures leads to a significant overall increase in expenditure. In total, around 300,000 THA procedures are carried out annually in the USA [94]. Up to 35% (105,000) of these are carried out in LVH [35, 95]. Given that the cost of 1 day of in-patient stay at a hospital in the USA is approximately $2500 [96], each additional day of stay is an additional annual expenditure of $262,500,000. This is excluding additional costs incurred due to the higher rates of SSI and other short-term (90-day post-THA) complications associated with having THA at LVH.

Although there is much debate on the influence of experience of the surgeon and outcomes [97, 98], lower mortality rate at 30 days, 90 days, and 1-year postoperatively in HVHs following hip arthroplasty may be because health care professionals including doctors and staff may have more experience and skill at HVH, with highly evolved and efficient processes of patient care (such as tailored diagnostic and treatment algorithms or guidelines), leading to better patient outcomes [99, 100]. In addition to this, the larger workforce and more system-level resources to use in patient care allow HVHs to tackle unanticipated complications at all stages of care [99].

The better outcomes observed in HVH may best be explained by two popular hypotheses which contribute to positive feedback. First, “practice makes perfect” which means hospitals develop more effective skills as they treat more patients [100] and second “selective referral” where physicians and hospitals with better outcomes receive more referrals subsequently acquiring larger volumes [101].

### Limitations

There are several caveats in our study. We were not able to review the intra-operative factors and the functional rehabilitation of patients for comparison between LVH and HVHs. This was because no studies have compared these variables among LVH and HVHs. Additionally, the hospital volume cutoff is not uniform across studies. Katz [35] used < 10 procedures per year as the cut-off, while Laucis [102] set < 100 cases per year as LVH. This could be due to the rising popularity of arthroplasty to treat end-stage osteoarthritis where the number of THA and TKA increased from 343,000 in 2000 to 851,000 in 2012 and is further on the rise. Hospitals now perform this procedure more frequently, and understandably, the cut-offs have been raised over time as observed in more recent studies. Studies have been conducted in 12 unique countries and variatons in volume thresholds may have been contributed by economical, logistic, and disease burden differences. Lastly, our review is based on observational studies. Conducting a randomized control trial (RCT) may have ethical and logistical barriers. Even after 30 years of the first study comparing the outcomes of THA in LVH and HVH, no RCT has been reported. There is a need for stronger evidence, including prospective cohorts, to re-visit this important topic using larger datasets to define objective volume-thresholds/benchmarks.

## Conclusion

Our analysis shows that total hip arthroplasties performed at low-volume hospitals have significantly higher surgical site infection rates, length of stay, cost of surgery, 90-day complications, and mortality rates (30 days, 90 days, and 1 year) compared with high-volume hospitals. Randomized controlled trials and prospective studies should be conducted to assess differences in functional outcomes and intra-operative factors between low-volume and high-volume hospitals using standardized cut-offs for low- and high-volume hospitals.

## Supplementary information

**Additional file 1.** Study characteristics.

**Additional file 2.** Covariate adjustment.

**Additional file 3.** Findings from each study (extracted data).

**Additional file 4: Table S4.** Hospital Volume Thresholds and Outcomes in Studies of Primary Total Hip Arthroplasty (pTHA).

**Additional file 5.** Comorbids reported across studies.

## Data Availability

The datasets used and/or analysed during the current study are available from the corresponding author on reasonable request.

## Abbreviations

ASA: American Society of Anesthesiologists
HVH: High-volume hospital
LOS: Length of stay
LVH: Low-volume hospital
RR: Risk ratio
SMD: Standard mean difference
SSI: Surgical site infection
THA: Total hip arthroplasty
