# Supplementary material for: Effect of hospital volume on outcomes of total hip arthroplasty: a systematic review and meta-analysis
Source: J Orthop Surg Res. 2019 Dec 27;14:468. doi: 10.1186/s13018-019-1531-0 (PMC6935169; doi:10.1186/s13018-019-1531-0)
Supplement: Supplementary file 3 — Additional file 3. Findings from each study (extracted data). [file 13018_2019_1531_MOESM3_ESM.docx]

| **Additional file 3: Quality Assessment of Included Studies** | | | | | | |
| --- | --- | --- | --- | --- | --- | --- |
| **Study** | **Are the main outcomes accurate, valid and reliable?** | **Are the main determinants accurate, valid and reliable?** | **Are statistical tests used appropriate for the aim of the study?** | **Were the losses of patients to follow-up taken into account?** | **Is the Confounding Variable adjustment accurate?** | **Total (/5)** |
| **Adelani, et al.^(^**[**^1^**](#_ENREF_1)**^)^** | 1 | 1 | 1 | 0 | 0 | 3 |
| **Boas, et al. ^(^**[**^2^**](#_ENREF_2)**^)^** | 1 | 1 | 1 | 0 | 0 | 3 |
| **Camberlin, et al.^(^**[**^3^**](#_ENREF_3)**^)^** | 1 | 1 | 1 | 0 | 1 | 4 |
| **Cossec, et al.^(^**[**^4^**](#_ENREF_4)**^)^** | 1 | 1 | 1 | 0 | 1 | 4 |
| **Doro, et al^(^**[**^5^**](#_ENREF_5)**^)^** | 1 | 1 | 1 | 0 | 1 | 4 |
| **Dy, et al. ^(^**[**^6^**](#_ENREF_6)**^)^** | 1 | 1 | 1 | 0 | 1 | 4 |
| **Espehaug, et al.^(^**[**^7^**](#_ENREF_7)**^)^** | 1 | 1 | 1 | 0 | 1 | 4 |
| **Glassou, et al.^(^**[**^8^**](#_ENREF_8)**^)^** | 1 | 1 | 1 | 0 | 1 | 4 |
| **Helkamaa, et al.^(^**[**^9^**](#_ENREF_9)**^)^** | 1 | 1 | 1 | 0 | 1 | 4 |
| **Huang, et al.^(^**[**^10^**](#_ENREF_10)**^)^** | 1 | 1 | 1 | 0 | 1 | 4 |
| **Hughes, et al. ^(^**[**^11^**](#_ENREF_11)**^)^** | 1 | 1 | 1 | 0 | 1 | 4 |
| **Jarvelin, et al.^(^**[**^12^**](#_ENREF_12)**^)^** | 1 | 1 | 1 | 0 | 1 | 4 |
| **Kaneko, et al.^(^**[**^13^**](#_ENREF_13)**^)^** | 1 | 1 | 1 | 0 | 1 | 4 |
| **Katz, et al. (2001)^(^**[**^14^**](#_ENREF_14)**^)^** | 1 | 1 | 1 | 0 | 1 | 4 |
| **Katz, et al. (2003)^(^**[**^15^**](#_ENREF_15)**^)^** | 1 | 1 | 1 | 0 | 1 | 4 |
| **Katz, et al. (2012)^(^**[**^16^**](#_ENREF_16)**^)^** | 1 | 1 | 1 | 0 | 0 | 3 |
| **Khatod, et al.^(^**[**^17^**](#_ENREF_17)**^)^** | 1 | 1 | 1 | 0 | 1 | 4 |
| **Khuri, et al.^(^**[**^18^**](#_ENREF_18)**^)^** | 1 | 1 | 1 | 1 | 1 | 5 |
| **Kreder, et al. (1997) ^(^**[**^19^**](#_ENREF_19)**^)^** | 1 | 1 | 1 | 1 | 1 | 5 |
| **Kreder, et al. (1998)^(^**[**^20^**](#_ENREF_20)**^)^** | 1 | 1 | 1 | 0 | 1 | 4 |
| **Laura, et al.^(^**[**^21^**](#_ENREF_21)**^)^** | 1 | 1 | 1 | 0 | 1 | 4 |
| **Losina, et al. ^(^**[**^22^**](#_ENREF_22)**^)^** | 1 | 1 | 1 | 1 | 1 | 5 |
| **Maceroli, et al.^(^**[**^23^**](#_ENREF_23)**^)^** | 1 | 1 | 1 | 1 | 1 | 5 |
| **Makela, et al.^(^**[**^24^**](#_ENREF_24)**^)^** | 1 | 1 | 1 | 1 | 1 | 5 |
| **Manley, et al.^(^**[**^25^**](#_ENREF_25)**^)^** | 1 | 1 | 1 | 0 | 1 | 4 |
| **Martineau, et al.^(^**[**^26^**](#_ENREF_26)**^)^** | 1 | 1 | 1 | 0 | 1 | 4 |
| **Meyer, et al.^(^**[**^27^**](#_ENREF_27)**^)^** | 1 | 1 | 1 | 1 | 1 | 5 |
| **Mitsuyasu, et al.^(^**[**^28^**](#_ENREF_28)**^)^** | 1 | 1 | 1 | 0 | 1 | 4 |
| **Muilwijk, et al.^(^**[**^29^**](#_ENREF_29)**^)^** | 1 | 1 | 1 | 0 | 1 | 4 |
| **Namba, et al.^(^**[**^30^**](#_ENREF_30)**^)^** | 1 | 1 | 1 | 1 | 0 | 4 |
| **Ong, et al.^(^**[**^31^**](#_ENREF_31)**^)^** | 1 | 1 | 1 | 0 | 1 | 4 |
| **Pablo, et al. ^(^**[**^32^**](#_ENREF_32)**^)^(2004)** | 1 | 1 | 1 | 1 | 1 | 5 |
| **Pablo, et al (2006) ^(^**[**^33^**](#_ENREF_33)**^)^** | 1 | 1 | 1 | 1 | 1 | 5 |
| **Pamilo, et al.^(^**[**^34^**](#_ENREF_34)**^)^** | 1 | 1 | 1 | 1 | 1 | 5 |
| **Paterson, et al.^(^**[**^35^**](#_ENREF_35)**^)^** | 1 | 1 | 1 | 1 | 1 | 5 |
| **Paxton, et al.^(^**[**^36^**](#_ENREF_36)**^)^** | 1 | 1 | 1 | 0 | 1 | 4 |
| **Ramkumar, et al.^(^**[**^37^**](#_ENREF_37)**^)^** | 1 | 1 | 1 | 0 | 0 | 4 |
| **Shi, et al.^(^**[**^38^**](#_ENREF_38)**^)^** | 1 | 1 | 1 | 1 | 1 | 5 |
| **Singh, et al.^(^**[**^39^**](#_ENREF_39)**^)^** | 1 | 1 | 1 | 1 | 1 | 5 |
| **Solomon, et al.^(^**[**^40^**](#_ENREF_40)**^)^** | 1 | 1 | 1 | 1 | 1 | 5 |
| **Soohoo, et al.^(^**[**^41^**](#_ENREF_41)**^)^** | 1 | 1 | 1 | 0 | 1 | 4 |
| **Styron, et al. ^(^**[**^42^**](#_ENREF_42)**^)^** | 1 | 1 | 1 | 0 | 1 | 4 |


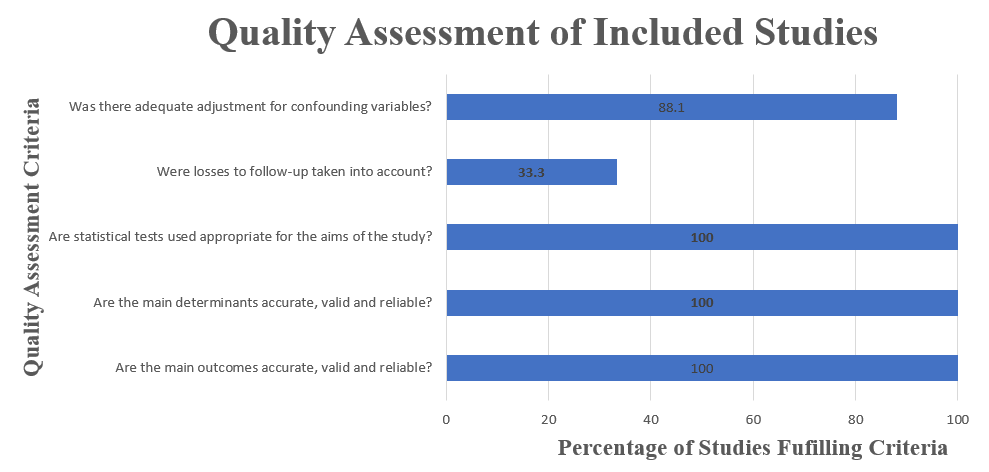


**References**

1. Adelani MA, Keller MR, Barrack RL, Olsen MA. The impact of hospital volume on racial differences in complications, readmissions, and emergency department visits following total joint arthroplasty. The Journal of arthroplasty. 2018;33(2):309-15. e20.

2. Boas R, Ensor K, Qian E, Hutzler L, Slover J, Bosco J. The relationship of hospital charges and volume to surgical site infection after total hip replacement. American journal of medical quality. 2015;30(3):283-8.

3. Cécile Camberlin M. Provider volume and short term complications after elective total hip replacement: an analysis of Belgian administrative data. Acta Orthopædica Belgica. 2011;77:311-9.

4. Le Cossec C, Colas S, Zureik M. Relative impact of hospital and surgeon procedure volumes on primary total hip arthroplasty revision: a nationwide cohort study in France. Arthroplasty today. 2017;3(3):176-82.

5. Doro C, Dimick J, Wainess R, Upchurch G, Urquhart A. Hospital volume and inpatient mortality outcomes of total hip arthroplasty in the United States. The Journal of arthroplasty. 2006;21(6):10-6.

6. Dy CJ, Bozic KJ, Pan TJ, Wright TM, Padgett DE, Lyman S. Risk factors for early revision after total hip arthroplasty. Arthritis care & research. 2014;66(6):907-15.

7. Espehaug B, Havelin LI, Engesaeter LB, Vollset SE. The effect of hospital-type and operating volume on the survival of hip replacements: a review of 39,505 primary total hip replacements reported to the Norwegian Arthroplasty Register, 1988-1996. Acta orthopaedica Scandinavica. 1999;70(1):12-8.

8. Glassou E, Hansen T, Mäkelä K, Havelin LI, Furnes O, Badawy M, et al. Association between hospital procedure volume and risk of revision after total hip arthroplasty: a population-based study within the Nordic Arthroplasty Register Association database. Osteoarthritis and cartilage. 2016;24(3):419-26.

9. Helkamaa T, Hirvensalo E, Huhtala H, Remes V. Patient injuries in primary total hip replacement: Nationwide analysis in Finland. Acta orthopaedica. 2016;87(3):209-17.

10. Huang C-S, Cheu Y-D, Ying J, Wei M-H. Association between provider volume and comorbidity on hospital utilization and outcomes of total hip arthroplasty among National Health Insurance enrollees. Journal of the Formosan Medical Association. 2011;110(6):401-9.

11. Hughes RG, Garnick DW, Luft HS, McPhee SJ, Hunt SS. Hospital volume and patient outcomes. The case of hip fracture patients. Med Care. 1988;26(11):1057-67. Epub 1988/11/01.

12. Järvelin J, Häkkinen U, Rosenqvist G, Remes V. Factors predisposing to claims and compensations for patient injuries following total hip and knee arthroplasty. Acta orthopaedica. 2012;83(2):190-6.

13. Kaneko T, Hirakawa K, Fushimi K. Relationship between peri-operative outcomes and hospital surgical volume of total hip arthroplasty in Japan. Health Policy. 2014;117(1):48-53.

14. Katz JN, Losina E, Barrett J, Phillips CB, Mahomed NN, Lew RA, et al. Association between hospital and surgeon procedure volume and outcomes of total hip replacement in the United States Medicare population. Jbjs. 2001;83(11):1622-9.

15. Katz JN, Phillips CB, Baron JA, Fossel AH, Mahomed NN, Barrett J, et al. Association of hospital and surgeon volume of total hip replacement with functional status and satisfaction three years following surgery. Arthritis and rheumatism. 2003;48(2):560-8. Epub 2003/02/07.

16. Katz JN, Wright EA, Wright J, Malchau H, Mahomed NN, Stedman M, et al. Twelve-year risk of revision after primary total hip replacement in the US Medicare population. The Journal of bone and joint surgery American volume. 2012;94(20):1825.

17. Khatod M, Cafri G, Namba RS, Inacio MC, Paxton EW. Risk factors for total hip arthroplasty aseptic revision. The Journal of arthroplasty. 2014;29(7):1412-7.

18. Khuri SF, Daley J, Henderson W, Hur K, Hossain M, Soybel D, et al. Relation of surgical volume to outcome in eight common operations: results from the VA National Surgical Quality Improvement Program. Annals of surgery. 1999;230(3):414.

19. Kreder HJ, Deyo RA, Koepsell T, Swiontkowski MF, Kreuter W. Relationship between the volume of total hip replacements performed by providers and the rates of postoperative complications in the state of Washington. Jbjs. 1997;79(4):485-94.

20. Kreder HJ, Williams JI, Jaglal S, Hu R, Axcell T, Stephen D. Are complication rates for elective primary total hip arthroplasty in Ontario related to surgeon and hospital volumes? A preliminary investigation. Canadian journal of surgery. 1998;41(6):431.

21. de Vries LM, Sturkenboom MC, Verhaar JA, Kingma JH, Stricker BH. Complications after hip arthroplasty and the association with hospital procedure volume: a nationwide retrospective cohort study on 50,080 total hip replacements with a follow-up of 3 months after surgery. Acta orthopaedica. 2011;82(5):545-52.

22. Losina E, Barrett J, Mahomed NN, Baron JA, Katz JN. Early failures of total hip replacement: effect of surgeon volume. Arthritis & Rheumatism: Official Journal of the American College of Rheumatology. 2004;50(4):1338-43.

23. Maceroli MA, Nikkel LE, Mahmood B, Elfar JC. Operative mortality after arthroplasty for femoral neck fracture and hospital volume. Geriatric orthopaedic surgery & rehabilitation. 2015;6(4):239-45.

24. Mäkelä KT, Häkkinen U, Peltola M, Linna M, Kröger H, Remes V. The effect of hospital volume on length of stay, re-admissions, and complications of total hip arthroplasty: a population-based register analysis of 72 hospitals and 30,266 replacements. Acta orthopaedica. 2011;82(1):20-6.

25. Manley M, Ong K, Lau E, Kurtz SM. Effect of volume on total hip arthroplasty revision rates in the United States Medicare population. Jbjs. 2008;90(11):2446-51.

26. Martineau P, Filion KB, Huk OL, Zukor DJ, Eisenberg MJ, Antoniou J. Primary hip arthroplasty costs are greater in low-volume than in high-volume Canadian hospitals. Clinical Orthopaedics and Related Research®. 2005;437:152-6.

27. Meyer E, Weitzel-Kage D, Sohr D, Gastmeier P. Impact of department volume on surgical site infections following arthroscopy, knee replacement or hip replacement. BMJ Qual Saf. 2011;20(12):1069-74.

28. Mitsuyasu S, Hagihara A, Horiguchi H, Nobutomo K. Relationship between total arthroplasty case volume and patient outcome in an acute care payment system in Japan. The Journal of arthroplasty. 2006;21(5):656-63.

29. Muilwijk J, van den Hof S, Wille JC. Associations between surgical site infection risk and hospital operation volume and surgeon operation volume among hospitals in the Dutch nosocomial infection surveillance network. Infection Control & Hospital Epidemiology. 2007;28(5):557-63.

30. Namba R, Inacio M, Paxton E. Risk factors associated with surgical site infection in 30 491 primary total hip replacements. The Journal of bone and joint surgery British volume. 2012;94(10):1330-8.

31. Ong K, Lau E, Manley M, Kurtz SM. Patient, hospital, and procedure characteristics influencing total hip and knee arthroplasty procedure duration. The Journal of arthroplasty. 2009;24(6):925-31.

32. Pablo Pd, Losina E, Phillips CB, Fossel AH, Mahomed N, Lingard EA, et al. Determinants of discharge destination following elective total hip replacement. Arthritis care & research. 2004;51(6):1009-17.

33. de Pablo P, Losina E, Mahomed N, Wright J, Fossel AH, Barrett JA, et al. Extent of followup care after elective total hip replacement. The Journal of Rheumatology. 2006;33(6):1159.

34. Pamilo KJ, Peltola M, Mäkelä K, Häkkinen U, Paloneva J, Remes V. Is hospital volume associated with length of stay, re-admissions and reoperations for total hip replacement? A population-based register analysis of 78 hospitals and 54,505 replacements. Archives of orthopaedic and trauma surgery. 2013;133(12):1747-55.

35. Paterson JM, Williams JI, Kreder HJ, Mahomed NN, Gunraj N, Wang X, et al. Provider volumes and early outcomes of primary total joint replacement in Ontario. Canadian journal of surgery. 2010;53(3):175.

36. Paxton EW, Inacio MC, Singh JA, Love R, Bini SA, Namba RS. Are there modifiable risk factors for hospital readmission after total hip arthroplasty in a US healthcare system? Clinical Orthopaedics and Related Research®. 2015;473(11):3446-55.

37. Ramkumar PN, Navarro SM, Frankel WC, Haeberle HS, Delanois RE, Mont MA. Evidence-based thresholds for the volume and length of stay relationship in total hip arthroplasty: outcomes and economies of scale. The Journal of arthroplasty. 2018;33(7):2031-7.

38. Shi H-Y, Chang J-K, Chiu H-C. Volume associations in total hip arthroplasty: a nationwide Taiwan population-based study. The Journal of arthroplasty. 2013;28(10):1834-8.

39. Singh JA, Kwoh CK, Boudreau RM, Lee GC, Ibrahim SA. Hospital volume and surgical outcomes after elective hip/knee arthroplasty: A risk‐adjusted analysis of a large regional database. Arthritis & Rheumatism. 2011;63(8):2531-9.

40. Solomon DH, Losina E, Baron JA, Fossel AH, Guadagnoli E, Lingard EA, et al. Contribution of hospital characteristics to the volume–outcome relationship: dislocation and infection following total hip replacement surgery. Arthritis & Rheumatism. 2002;46(9):2436-44.

41. SooHoo NF, Farng E, Lieberman JR, Chambers L, Zingmond DS. Factors that predict short-term complication rates after total hip arthroplasty. Clinical Orthopaedics and Related Research®. 2010;468(9):2363-71.

42. Styron JF, Koroukian SM, Klika AK, Barsoum WK. Patient vs provider characteristics impacting hospital lengths of stay after total knee or hip arthroplasty. The Journal of arthroplasty. 2011;26(8):1418-26.e262. Epub 2011/01/28.
