# Supplementary material for: Effect of hospital volume on outcomes of total hip arthroplasty: a systematic review and meta-analysis
Source: J Orthop Surg Res. 2019 Dec 27;14:468. doi: 10.1186/s13018-019-1531-0 (PMC6935169; doi:10.1186/s13018-019-1531-0)
Supplement: Supplementary file 4 — Additional file 4: Table S4. Hospital Volume Thresholds and Outcomes in Studies of Primary Total Hip Arthroplasty (pTHA). [file 13018_2019_1531_MOESM4_ESM.docx]

| **Table S4: Hospital Volume Thresholds and Outcomes in Studies of Primary Total Hip Arthroplasty (pTHA)** | | | | | | | | | |
| --- | --- | --- | --- | --- | --- | --- | --- | --- | --- |
| **Study** | **Hospital Volume Thresholds for pTHA** | | | | | **Outcomes** | **Significance** | **Outcome Results** | **OR/ RR/HR/Rate/Others [95% CI]** |
|  | **Very Low** | **Low** | **Medium** | **High** | **Very High** |  |  |  |  |
| **Adelani, et al. ^(^**[**^1^**](#_ENREF_1)**^)^** | - | 1-36 | 37-121 | 122-275 | 282-3703 | **30-days Readmission**   - Low Volume Hospitals - Medium Volume Hospitals - High Volume Hospitals - Very High Volume Hospitals     **90-day Readmission**   - Low Volume Hospitals - Medium Volume Hospitals - High Volume Hospitals - Very High Volume Hospitals | **30-days Readmission**   - NS - NS - NS - Reference   **90-day Readmission**   - NS - *S (P <0.05)* - NS - Reference |  | **30-days Readmission RR**   - 1.01[0.90-1.14] - 1.08 [0.99-1.18] - 1.00 [0.93-1.09] - Reference   **90-day Readmission RR**   - 1.07 [0.97-1.18] - 1.10 [1.02-1.18] - 1.01 [0.95-1.07] - Reference |
| **Boas, et al. ^(^**[**^2^**](#_ENREF_2)**^)^** | - | 26-99 | >99 | >299 | >599 | **Total Hospital Charges**   - Low Volume Hospitals - Very High Volume Hospitals     **Surgical Site Infection**   - Low Volume Hospitals - Very High Volume Hospitals | **Total Hospital Charges**   - Reference - *S (P* < .0001)   **Surgical Site Infection**   - Reference - *S (P=0.0030)* | **Charges ($)**   - 43,609.6 - 52,671.9   **-** | -  **Surgical Site Infection Adjusted Rate /100**   - 1.26 (1.08-1.44) - *0.68 (0.50-0.85)* |
| **Camberlin, et al.^(^**[**^3^**](#_ENREF_3)**^)^** | - | <60 | 60-110 | >110 | - | **90-day Short Term Complications**   - Low Volume Hospitals - Medium Volume Hospitals - High Volume Hospitals | - NS - NS - Reference | **Percentage**   - 4.1 - 4.1 - 2.9 | **Adjusted OR**   - 0.98 (0.68, 1.43) - 1.19 (0.87, 1.64) - Reference |
| **Cossec, et al.**  **^(^**[**^4^**](#_ENREF_4)**^)^** | - | < 7  / month | 7-15  / month | > 15  / month | - | **THA Revision**   - Low Volume Hospitals - Medium Volume Hospitals - High Hospitals | - NS (P = 0.39) - NS - Reference |  | **Adjusted OR**   - 1.11 (0.92-1.34) - 1.07 (0.95- 1.22) - Reference |
| **Doro, et al. ^(^**[**^5^**](#_ENREF_5)**^)^** | - | 1-40 | 41-79 | 80-139 | ≥140 | **Inpatient Mortality**   - Low Volume Hospitals - Medium Volume Hospitals - High Volume Hospitals - Very High Volume Hospitals   **Prolonged Length of Stay**   - Low Volume Hospitals - Medium Volume Hospitals - High Volume Hospitals - Very High Volume Hospitals   **Discharge to ECF**   - Low Volume Hospitals - Medium Volume Hospitals - High Volume Hospitals - Very High Volume Hospitals | **Inpatient Mortality**   - NS (P =.003) - NS (P =.096) - NS (P =.328) - Reference   **Prolonged Length of Stay**   - *S (P <.001)* - NS (P =.002) - *S (P <.001)* - Reference   **Discharge to ECF**   - *S (P <.001)* - NS (P =.002) - *S (P <.001)* - Reference | **Percentage**   - 0.28 - 0.23 - 0.19 - 0.16   **Percentage**   - *24* - 17 - *14* - 14   **Percentage**   - *42* - 40 - *37* - 36 | **Inpatient Mortality Adjusted OR**   - 1.3 (0.79-2.03) - 1.5 (0.94-2.28) - 1.9 (1.23-2.78) - Reference   **Prolonged Length of Stay Adjusted OR**   - *1.2 (1.11-1.21)* - 1.1 (1.03 -1.12) - *0.9 (0.85- 0.93)* - Reference   **Discharge to ECF Adjusted OR**   - *0.9 (0.83 -0.93)* - 1.0 (0.90- 1.01) - *0.9 (0.83 -0.93)* - Reference |
| **Dy, et al. ^(^**[**^6^**](#_ENREF_6)**^)^** | - | <201 | 201-400 | <400 | - | **Septic Revision**   - Low Volume Hospitals - Medium Volume Hospitals - High Volume Hospitals   **Aseptic Revision**   - Low Volume Hospitals - Medium Volume Hospitals - High Volume Hospitals | **Septic Revision**   - NS (P=0.342) - NS (P=0.488) - Reference   **Aseptic Revision**   - *S (P=0.001)* - *S (P=0.001)* - Reference | - | **Septic Revision Adjusted HR**   - 1.22 (0.81–1.84) - 1.14 (0.75–1.74) - Reference   **Aseptic Revision Adjusted HR**   - *1.33 (1.16–1.52)* - *1.42 (1.24–1.64)* - Reference |
| **Espehaug, et al.^(^**[**^7^**](#_ENREF_7)**^)^** | ≤35 | 36-52 | 53-81 and 82-132 | 133-213 | >213 | **Revision Rate (Cemented)**   - Very Low Volume Hospitals - Low Volume Hospitals - Low Med. Volume Hospitals - High Med. Volume Hospitals - High Volume Hospitals - Very High Volume Hospitals   **Revision Rate (Uncemented)**   - Very Low Volume Hospitals - Low Volume Hospitals - Low Med. Volume Hospitals - High Med. Volume Hospitals - High Volume Hospitals - Very High Volume Hospitals | **Revision Rate (Cemented)**   - Reference - NS=0.76 - NS=0.95 - NS=0.54 - NS=0.08 - NS=0.08   **Revision Rate (Uncemented)**   - Reference - NS=0.02 - *S (P=0.004)* - *S (P=0.004)* - *S (P=0.04)* - *S (P<0.001)* | **Percentage**   - 3.8 - 3.8 - 3.9 - 3.9 - 4.6 - 5.1   **Percentage**   - 12.2 - 9.1 - *8.1* - *7.5* - *9.8* - *5.0* | **Revision Rate (Cemented) Adjusted RR**   - Reference - 1.0 (0.82-1.32) - 1.0 (0.79-1.25) - 1.1 (0.85-1.35) - 1.2 (0.98-1 59) - 1.3 (0.97-1.82)   **Revision Rate (Uncemented) Adjusted RR**   - Reference - 0.7 (0.48-0.94) - *0.6 (0.42-0.85)* - *0.6 (0.41-0.84)* - *0.6 (0.38-0.97)* - *0.4 (0.28-0.66)* |
| **Glassou, et al.^(^**[**^8^**](#_ENREF_8)**^)^** | 0-50 | 51-100 | 101-200 | 201-300 | >300 | **Cemented Revisions 0-1 years**   - Very Low Volume Hospitals - Low Volume Hospitals - Medium Volume Hospitals - High Volume Hospitals - Very High Volume Hospitals   **Cemented Revisions 1-2 years**   - Very Low Volume Hospitals - Low Volume Hospitals - Medium Volume Hospitals - High Volume Hospitals - Very High Volume Hospitals   **Cemented Revisions 2-5 years**   - Very Low Volume Hospitals - Low Volume Hospitals - Medium Volume Hospitals - High Volume Hospitals - Very High Volume Hospitals   **Cemented Revisions 5-10 years**   - Very Low Volume Hospitals - Low Volume Hospitals - Medium Volume Hospitals - High Volume Hospitals - Very High Volume Hospitals   **Cemented Revisions 10-15 years**   - Very Low Volume Hospitals - Low Volume Hospitals - Medium Volume Hospitals - High Volume Hospitals - Very High Volume Hospitals   **Uncemented Revisions 0-1 years**   - Very Low Volume Hospitals - Low Volume Hospitals - Medium Volume Hospitals - High Volume Hospitals - Very High Volume Hospitals   **Uncemented Revisions 1-2 years**   - Very Low Volume Hospitals - Low Volume Hospitals - Medium Volume Hospitals - High Volume Hospitals - Very High Volume Hospitals   **Uncemented Revisions 2-5 years**   - Very Low Volume Hospitals - Low Volume Hospitals - Medium Volume Hospitals - High Volume Hospitals - Very High Volume Hospitals   **Uncemented Revisions 5-10 years**   - Very Low Volume Hospitals - Low Volume Hospitals - Medium Volume Hospitals - High Volume Hospitals - Very High Volume Hospitals   **Uncemented Revisions 10-15 years**   - Very Low Volume Hospitals - Low Volume Hospitals - Medium Volume Hospitals - High Volume Hospitals - Very High Volume Hospitals | **Cemented Rev. 0-1 years**   - Reference - NS (P= 0.42) - *S (P=0.03)* - NS (P= 0.46) - NS (P= 0.67)   **Cemented Rev. 1-2 years**   - Reference - *S (P= 0.00)* - *S (P= 0.00)* - *S (P= 0.03)* - *S (P= 0.03)*   **Cemented Rev. 2-5 years**   - Reference - S (P= 0.00) - S (P= 0.00) - S (P= 0.00) - S (P= 0.00)   **Cemented Rev. 5-10 years**   - Reference - *S (P= 0.01)* - *S (P= 0.02)* - *S (P= 0.02)* - *S (P= 0.00)*   **Cemented Rev. 10-15 years**   - Reference - *S (P= 0.02)* - *S (P= 0.02)* - NS (P= 0.38) - *S (P= 0.00)*   **Uncemented Rev. 0-1 years**   - Reference - NS (P=0.65) - NS (P=0.97) - NS (P=0.50) - NS (P=0.51)   **Uncemented Rev. 1-2 years**   - Reference - NS (P=0.07) - NS (P=0.09) - NS (P=0.35) - NS (P=0.31)   **Uncemented Rev. 2-5 years**   - Reference - NS (P=0.92) - NS (P=0.21) - NS (P=0.05) - NS (P=0.68)   **Uncemented Rev. 5-10 years**   - Reference - NS (P=0.29) - NS (P=0.17) - NS (P=0.03) - NS (P=0.78)   **Uncemented Rev. 10-15 years**   - Reference - NS (P=0.17) - NS (P=0.13) - *S (P=0.08)* - NS (P=0.05) | **Number (%)**   - 224 (0.96) - 640 (0.93) - *825 (0.79)* - 304 (0.88) - 326 (1.03)   **Number (%)**   - *115 (0.66)* - *331 (0.48)* - *446 (0.43)* - *163 (0.47)* - *115 (0.36)*   **Number (%)**   - *323 (1.39)* - *729 (0.06)* - *973 (0.93)* - *285 (0.83)* - *183 (0.58)*   **Number (%)**   - *461 (1.98)* - *1,132 (1.64)* - *1,449 (1.38)* - *366 (1.06)* - *131 (0.41)*   **Number (%)**   - 266 (1.14) - *666 (0.97)* - *714 (0.68)* - 153 (0.44) - *30 (0.09)*   **Number (%)**   - 151 (1.92) - 397 (1.86) - 612 (1.92) - 331 (2.08) - 361 (1.76)   **Number (%)**   - 42 (0.53) - 164 (0.77) - 211 (0.66) - 85 (0.53) - 102 (0.50)   **Number (%)**   - 97 (1.23) - 263 (1.23) - 269 (0.84) - 89 (0.56) - 137 (0.67)   **Number (%)**   - 165 (2.09) - 369 (1.73) - 283 (0.89) - 69 (0.43) - 42 (0.20)   **Number (%)**   - 131 (1.66) - 283 (1.32) - 182 (0.57) - *28 (0.18)* - 11 (0.05) | **Cemented Rev. 0-1 years Adjusted RR**   - Reference - 0.93 (0.77-1.11) - *0.79 (0.64-0.98)* - 0.90 (0.69-1.18) - 1.05 (0.84-1.32)   **Cemented Rev. 1-2 years Adjusted RR**   - Reference - *0.72 (0.58-0.90)* - *0.64 (0.50-0.82)* - *0.74 (0.56-0.98)* - *0.74 (0.56-0.98)*   **Cemented Rev. 2-5 years Adjusted RR**   - Reference - *0.72 (0.59-0.87)* - *0.68 (0.55-0.83)* - *0.66 (0.52-0.85)* - *0.61 (0.46-0.81)*   **Cemented Rev. 5-10 years Adjusted RR**   - Reference - *0.79 (0.65-0.95)* - *0.76 (0.61-0.95)* - *0.74 (0.57-0.96)* - *0.57 (0.46-0.71)*   **Cemented Rev. 10-15 years Adjusted RR**   - Reference - *0.75 (0.60-0.95)* - *0.74 (0.58-0.96)* - 0.85 (0.60-1.22) - *0.43 (0.34-0.54)*   **Uncemented Rev. 0-1 years Adjusted RR**   - Reference - 0.95 (0.75-1.20) - 1.00 (0.77-1.28) - 1.11 (0.82-1.49) - 0.92 (0.72-1.18)   **Uncemented Rev. 1-2 years Adjusted RR**   - Reference - 1.41 (0.98-2.05) - 1.36 (0.95-1.95) - 1.23 (0.80-1.87) - 1.22 (0.83-1.80)   **Uncemented Rev. 2-5 years Adjusted RR**   - Reference - 0.96 (0.72-1.28) - 0.82 (0.62-1.09) - 0.71 (0.51-0.99) - 1.11 (0.65-1.92)   **Uncemented Rev. 5-10 years Adjusted RR**   - Reference - 0.82 (0.59-1.15) - 0.77 (0.54-1.09) - 0.59 (0.38-0.93) - 0.87 (0.56-1.35)   **Uncemented Rev. 10-15 years Adjusted RR**   - Reference - 0.74 (0.57-1.02) - 0.71 (0.50-1.01) - *0.62 (0.40-0.95)* - 1.70 (0.75-3.89) |
| **Helkamaa, et al.^(^**[**^9^**](#_ENREF_9)**^)^** | - | <100 | 100-199 | 200-399 | <400 | **Filed Claims**   - Low Volume Hospitals - Medium Volume Hospitals - High Volume Hospitals - Very High Volume Hospitals   **Compensated Claims**   - Low Volume Hospitals - Medium Volume Hospitals - High Volume Hospitals - Very High Volume Hospitals | **Filed Claims**   - S (P <0.05) - S (P <0.05) - S (P <0.05) - Reference   **Compensated Claims**   - S (P <0.05) - S (P <0.05) - S (P <0.05) - Reference | **Number**   - 140 - 205 - 98 - 120   **Number**   - 60 - 106 - 40 - 44 | **Filed Claims RR**   - 1.8 (1.4–2.2) - 1.9 (1.5–2.4) - 1.6 (1.3–2.1) - Reference   **Compensated Claims RR**   - 2.1 (1.4–2.4) - 2.7 (1.9–3.8) - 1.8 (1.2–2.8) - Reference |
| **Huang, et al.^(^**[**^10^**](#_ENREF_10)**^)^** | - | Lower 40^th^ Percentile | 40-80^th^ percentile | Top 80th percentile |  | **Length of Stay**   - Low Volume Hospitals - Medium Volume Hospitals - High Volume Hospitals   **Total Charges**   - Low Volume Hospitals - Medium Volume Hospitals - High Volume Hospitals   **Perioperative Complications**   - Low Volume Hospitals - Medium Volume Hospitals - High Volume Hospitals | **Length of Stay**   - Reference - NS 0.286 - NS 0.344     **Total Charges**   - Reference - NS 0.246 - NS 0.894   **Perioperative Complications**   - Reference - NS 0.669 - NS 0.542 | **Days (x̅± SD)**   - 8.74 ± 3.25 - 9.00 ± 3.93 - 7.41 ± 3.21   **NT$ (x̅ ±SD)**   - 111,089 ± 14,565 - 112,309 ± 17, 287 - 109,864 ± 18,002   **Number (%)**   - 6 (2.21) - 37 (1.93) - 98 (1.37) | -      -  **Perioperative Complications**   - Reference - 0.80 (0.25–2.53) - 0.67 (0.19–2.40) |
| **Hughes, et al ^(^**[**^11^**](#_ENREF_11)**^)^** |  | Low Volume |  | High Volume |  | **Bad Outcomes (>90^th^ percentile LOS and Mortality)**   - Low Volume Hospitals - High Volume Hospitals | - Reference - *S (P < 0.01)* | **(Expected vs. Observed)**   - Reference - -0.4782 (0.0862) |  |
| **Jarvelin, et al.^(^**[**^12^**](#_ENREF_12)**^)^** | - | <200 | 200-300 | >300 |  | **Patients Filing Claims**   - Low Volume Hospitals - Medium Volume Hospitals - High Volume Hospitals   **Claim Received: Treatment injury**   - Low Volume Hospitals - Medium Volume Hospitals - High Volume Hospitals   **Claim Received: Infection injury**   - Low Volume Hospitals - Medium Volume Hospitals - High Volume Hospitals | **Patients Filing Claims**   - Reference - *S (P=0.04)* - NS (P=0.9)   **Claim Rec.: Treatment injury**   - Reference - NS (P=0.9) - NS (P=1.0)   **Claim Rec.: Infection injury**   - Reference - NS (P=0.9) - NS (P=0.8) | **Number**   - 297 - *90* - 21 | **Patients Filing Claims**   - Reference - *1.29 (1.01–1.64)* - 1.02 (0.65–1.62)   **Claim Rec.: Treatment injury**   - Reference - 0.97 (0.58–1.63) - 1.03 (0.39–2.69)   **Claim Rec.: Infection injury**   - Reference - 0.95 (0.44–2.05) - 0.86 (0.18–4.14) |
| **Kaneko, et al. ^(^**[**^13^**](#_ENREF_13)**^)^** | - | 0-16 | 17-33 | 34-54 | >55 | **Dislocation**   - Low Volume Hospitals - Medium Volume Hospitals - High Volume Hospitals - Very High Volume Hospitals   **Fracture**   - Low Volume Hospitals - Medium Volume Hospitals - High Volume Hospitals - **V**ery High Volume Hospitals   **Pulmonary Embolism**   - Low Volume Hospitals - Medium Volume Hospitals - High Volume Hospitals - **V**ery High Volume Hospitals   **Infection**   - Low Volume Hospitals - Medium Volume Hospitals - High Volume Hospitals - Very High Volume Hospitals | **Dislocation**   - Reference - *S (P< 0.05)* - *S (P< 0.05)* - *S (P< 0.05)*   **Fracture**   - Reference - NS (P> 0.05) - NS (P> 0.05) - NS (P> 0.05)   **Pulmonary Embolism**   - Reference - *S (P< 0.05)* - NS (P> 0.05) - NS (P> 0.05)   **Infection**   - Reference - NS (P> 0.05) - *S (P< 0.05)* - *S (P< 0.05)* |  | **Dislocation Adjusted OR**   - Reference - *0.558(0.352–0.870)* - *0.435(0.250–0.724)* - *0.321(0.167–0.572)*   **Fracture Adjusted OR**   - Reference - 0.457(0.175–1.075) - 0.725(0.305–1.617) - 0.587(0.224–1.389)   **Pulmonary Embolism Adjusted OR**   - Reference - *0.491(0.228–0.995)* - 0.487(0.212–1.029) - 1.145(0.614–2.125)   **Infection Adjusted OR**   - Reference - 1.019(0.563–1.846) - *3.148(1.937–5.295)* - *0.123(0.020–0.421)* |
| **Katz, et al. 2001 ^(^**[**^14^**](#_ENREF_14)**^)^** | 1-10 | 11-25 | 26-50 | 51-100 | >100 | **90-day Dislocation**   - Very Low Volume Hospitals - Low Volume Hospitals - Medium Volume Hospitals - High Volume Hospitals - Very High Volume Hospitals   **90-day Deep Infection**   - Very Low Volume Hospitals - Low Volume Hospitals - Medium Volume Hospitals - High Volume Hospitals - Very High Volume Hospitals   **90-day Pulmonary Embolism**   - Very Low Volume Hospitals - Low Volume Hospitals - Medium Volume Hospitals - High Volume Hospitals - Very High Volume Hospitals   **90- day Mortality**   - Very Low Volume Hospitals - Low Volume Hospitals - Medium Volume Hospitals - High Volume Hospitals - Very High Volume Hospitals | **90-day Dislocation**   - Reference - NS - *S* - *S* - NS   **90-day Deep Infection**   - Reference - NS - *S* - NS - NS   **90-day Pulmonary Embolism**   - Reference - NS - NS - NS - NS   **90- day Mortality**   - Reference - NS - *S* - *S* - *S* | **Percentage**   - 4.4% - 3.8% - *2.9%* - *2.5%* - 2.2%   **Percentage**   - 0.1% - 0.3% - *0.2%* - 0.2% - 0.1%   **Percentage**   - 1.1% - 1.0% - 1.0% - 0.8% - 0.8%   **Percentage**   - 1.3% - 1.0% - *0.9%* - *0.9%* - *0.7%* | **90-day Dislocation Adjusted OR**   - Reference - 0.096(0.82,1.17 - *0.79(0.67, 0.93)* - *0.72(0.60, 0.87*) - 0.77(0.58, 1.03)   **90-day Deep Infection Adjusted OR**   - Reference - 0.84(0.52, 1.37) - *0.56(0.33, 0.96)* - 0.74(0.42, 1.32) - 0.52(0.22, 1.22)   **90-day Pulmonary Embolism Adjusted OR**   - Reference - 0.86(0.64, 1.15) - 0.89(0.66, 1.21) - 0.83(0.60, 1.14) - 0.79(0.51, 1.23)   **90- day Mortality Adjusted OR**   - Reference - 0.82(0.62, 1.07) - *0.72(0.54, 0.95)* - *0.68(0.51, 0.92)* - *0.58(0.38, 0.89)* |
| **Katz, et al. 2003 ^(^**[**^15^**](#_ENREF_15)**^)^** | - | 1-12 | 13-100 | >100 | - | **Harris Hip score in lowest 10%**   - Low Volume Hospitals - Medium Volume Hospitals - High Volume Hospitals   **Satisfaction Score < 50**   - Low Volume Hospitals - Medium Volume Hospitals - High Volume Hospitals | **Harris Hip Score Lowest (10%)**   - NS (P>0.05) - NS (P>0.05) - Reference   **Satisfaction Score < 50**   - *S (P<0.01)* - NS (P>0.05) - Reference | **Percentage**   - 13.1 - 10.0 - 7.8   **Percentage**   - *13.8* - 8.8 - 6.9 | **Harris Hip Score in Lowest 10% Adjusted OR**   - 1.29 (0.64–2.62) - 1.14 (0.63–2.06) - Reference   **Satisfaction Score < 50 Adjusted OR**   - *2.06 (1.15–3.69)* - 1.22 (0.70–1.13) - Reference |
| **Katz, et al. 2012 ^(^**[**^16^**](#_ENREF_16)**^)^** | - | 1-25 | 26-50 | >50 |  | **Revision**   - Low Volume Hospitals - Medium Volume Hospitals - High Volume Hospitals | **Revision**   - NS (P>0.05) - NS (P >0.05) - Reference |  | **Revision Hazard Ratio**   - 1.14 (1.07, 1.23) - (0.94, 1.10) - Reference |
| **Khatod, et al. ^(^**[**^17^**](#_ENREF_17)**^)^** |  | < 75 | - | ≥ 75 | - | **Aseptic Revision**   - Low Volume Hospitals - Hospital Volume Hospitals | **Aseptic Revision**   - NS 0.769 - Reference | - | **HR**   - 1.10 (0.58 -2.07) - Reference |
| **Khuri, et al. ^(^**[**^18^**](#_ENREF_18)**^)^** | - | 0-10 | 11-16 | 17-22 | 23-55 | **Mortality (30 day)**   - Low Volume Hospitals - Medium Volume Hospitals - High Volume Hospitals - Very High Volume Hospitals | NS (P = 0.52) |  | **Observed Mortality/Expected Mortality Ratio**   - 0.80 (± 2.9) - 0.66 (± 1.0) - 0.98 (± 0.9) - 1.43 (± 1.2) |
| **Kreder, et al. 1997 ^(^**[**^19^**](#_ENREF_19)**^)^** | - | < 40^th^ Percentile | 40^th^-80^th^ Percentile | > 80^th^ Percentile | - | **Death < 3months**   - Low Volume Hospitals - Medium Volume Hospitals - High Volume Hospitals   **Death <1 year**   - Low Volume Hospitals - Medium Volume Hospitals - High Volume Hospitals   **Infection <3months**   - Low Volume Hospitals - Medium Volume Hospitals - High Volume Hospitals   **Infection <1 year**   - Low Volume Hospitals - Medium Volume Hospitals - High Volume Hospitals   **Revision <3months**   - Low Volume Hospitals - Medium Volume Hospitals - High Volume Hospitals   **Revision <1 year**   - Low Volume Hospitals - Medium Volume Hospitals - High Volume Hospitals   **Inpatient Complications**   - Low Volume Hospitals - Medium Volume Hospitals - High Volume Hospitals   **Difference is Hospital Costs**   - Low Volume Hospitals - Medium Volume Hospitals - High Volume Hospitals   **Difference in LOS**   - Low Volume Hospitals - Medium Volume Hospitals - High Volume Hospitals | **Death < 3months**   - NS - NS - Reference   **Death <1 year**   - NS - *S < 0.05* - Reference   **Infection <3months**   - NS - NS - Reference   **Infection <1 year**   - NS - NS - Reference   **Revision <3months**   - NS - NS - Reference   **Revision <1 year**   - NS - NS - Reference   **Inpatient Complications**   - NS - NS - Reference   **Difference is hospital charge**   - *S <0.05* - *S <0.05* - Reference   **Difference in LOS**   - *S <0.05* - NS - Reference | **Percentage**   - 2.1 - 0.9 - 0.5   **Percentage**   - 3.2 - *2.0* - 1.3   **Percentage**   - 1.1 - 0.7 - 0.3   **Percentage**   - 1.1 - 1.2 - 0.6   **Percentage**   - 1.8 - 0.7 - 0.5   **Percentage**   - 3.2 - 1.9 - 1.6   **Percentage**   - NS - NS - Reference   **Avg. Cost ($)**   - 13,294 - 13,103 - 11,729   **Avg. Days**   - 6.99 - 7.76 - 7.85 | **Death < 3months Adjusted OR**   - 0.9 (0.2 to 3.9) - 1.8 (1.0 to 3.3) - Reference   **Death <1 year Adjusted OR**   - 1.1 (0.5 to 2.9) - 1.7 (1.1 to 2.4) - Reference   **Infection <3months Adjusted OR**   - (0.2 to 4.2) - 1.2 (0.7 to 2.4) - Reference   **Infection <1 year Adjusted OR**   - 0.9 (0.2 to 4.0) - 1.3 (0.8 to 2.1) - Reference   **Revision <3months Adjusted OR**   - 1.8 (0.6 to 5.1) - 1.1 (0.6 to 2.1) - Reference   **Revision <1 year Adjusted OR**   - 0.9 (0.4 to 2.1) - 1.0 (0.7 to 1.4) - Reference   **Inpatient Complications Adjusted OR**   - 0.8 (0.4 to 1.4) - 0.9 (0.7 to 1.1) - Reference   **Difference is Hospital Cost ($)**   - *2630 (1758 to 3501)* - *1838 (1129 to 2546)* - Reference   **Difference in LOS Adjusted OR**   - *-1.0 (-1.4 to -0.6)* - -0.2 (-0.5 to 0.1) - Reference |
| **Kreder, et al. (1998) ^(^**[**^20^**](#_ENREF_20)**^)^** | - | < 40^th^ Percentile | 40^th^-80^th^ Percentile | > 80^th^ Percentile | - | **Readmission for Hip Infection <1Y**   - Low Volume Hospitals - Medium Volume Hospitals - High Volume Hospitals   **Readmission for Hip Revision <3Y**   - Low Volume Hospitals - Medium Volume Hospitals - High Volume Hospitals   **Readmission for Hip Revision <1Y**   - Low Volume Hospitals - Medium Volume Hospitals - High Volume Hospitals   **Patient Death <3M**   - Low Volume Hospitals - Medium Volume Hospitals - High Volume Hospitals   **Patient Death <1Y**   - Low Volume Hospitals - Medium Volume Hospitals - High Volume Hospitals   **Inpatient Complications**   - Low Volume Hospitals - Medium Volume Hospitals - High Volume Hospitals   **Length of Stay**   - Low Volume Hospitals - Medium Volume Hospitals - High Volume Hospitals | **Readmission Hip Infection <1Y**   - NS (P > 0.05) - NS (P > 0.05) - Reference   **Readmission Hip Revision <3Y**   - NS (P > 0.05) - NS (P > 0.05) - Reference   **Readmission Hip Revision <1Y**   - NS (P > 0.05) - NS (P > 0.05) - Reference   **Patient Death <3M**   - NS (P > 0.05) - NS (P > 0.05) - Reference   **Patient Death <1Y**   - NS (P > 0.05) - NS (P > 0.05) - Reference   **Inpatient Complications**   - NS (P > 0.05) - NS (P > 0.05) - Reference   **Length of Stay**   - NS (P > 0.05) - NS (P > 0.05) - Reference |  | **Readmission Hip Infection <1Y**   - 1.1 (0.4, 3.2) - 1.2 (0.6, 2.6) - Reference   **Readmission Hip Revision <3Y**   - 0.5 (0.2, 1.2) - 0.8 (0.4, 1.3) - Reference   **Readmission Hip Revision <1Y**   - 0.4 (0.1, 1.8) - 0.9 (0.4, 1.9) - Reference   **Patient Death <3M**   - 0.5 (0.1, 2.7) - 0.7 (0.2, 2.1) - Reference   **Patient Death <1Y**   - 1.0 (0.4, 2.6) - 1.0 (0.5, 2.1) - Reference   **Inpatient Complications**   - 0.7 (0.4, 1.2) - 1.0 (0.7, 1.4) - Reference   **Length of Stay**   - 0.0 (−0.9, 0.7) - −0.5 (−1.0, −0.0) - Reference |
| **Laura, et al.^(^**[**^21^**](#_ENREF_21)**^)^** | <100 | 100-199 | 200-299 | 300-399 | >400 | **Mortality (Index Admission)**   - Very Low Volume Hospitals - Low Volume Hospitals - Medium Volume Hospitals - High Volume Hospitals - Very High Volume Hospitals   **Complication (Index Admission)**   - Very Low Volume Hospitals - Low Volume Hospitals - Medium Volume Hospitals - High Volume Hospitals - Very High Volume Hospitals   **Readmission**   - Very Low Volume Hospitals - Low Volume Hospitals - Medium Volume Hospitals - High Volume Hospitals - Very High Volume Hospitals | **Mortality (Ind. Admission)**   - NS (P > 0.01) - NS (P > 0.01) - NS (P > 0.01) - NS (P > 0.01) - Reference   **Complication (Ind. Admission)**   - *S (P < 0.01)* - NS (P > 0.01) - NS (P > 0.01) - NS (P > 0.01) - Reference   **Readmission**   - *S (P < 0.01)* - NS (P > 0.01) - NS (P > 0.01) - NS (P > 0.01) - Reference |  | **Mortality (Ind. Admission) Adjusted OR**   - 0.17 (0.02–1.2) - 0.76 (0.44–1.3) - 1.1 (0.6–2) - 0.8 (0.44–1.5) - Reference   **Complication (Ind. Admission) Adjusted OR**   - *1.9 (1.4–2.6)* - 1.8 (1.5–2.3) - 1.9 (1.5–2.4) - 2.2 (1.7–2.7) - Reference   **Readmission Adjusted OR**   - *0.79 (0.71–0.87)* - 1.1 (0.87–1.4) - 0.94 (0.82–1.1) - 1 (0.88–1.2) - Reference |
| **Losina, et al. ^(^**[**^22^**](#_ENREF_22)**^)^** |  | 1-25 | 26-50 | 51-100 | >100 | **4 Year Revision THA**   - Low Volume Hospitals - Medium Volume Hospitals - High Volume Hospitals - Very High Volume Hospitals | **4 Year Revision THA**   - Reference - NS (P>0.05) - S (P<0.05) - S (P<0.05) |  | **4 Year Revision THA HR (95% CI)**   - Reference - 0.81 (0.73–0.91) - 0.89 (0.78–1.00) - 0.96 (0.76–1.21) |
| **Maceroli, et al.^(^**[**^23^**](#_ENREF_23)**^)^** |  | Bottom Quartile |  | Top Quartile |  | **Mortality within 1 Year**   - Low Volume Hospitals - High Volume Hospitals | - Reference - *S (P=0.008)* | **Percentage**   - 18.12% - *7.60%* | **Mortality within 1 Year Hazard Ratio (95% CI)**   - Reference - *0.553* |
| **Makela, et al.^(^**[**^24^**](#_ENREF_24)**^)^** |  | 1-50 | 51-100 | 151-300 | >300 | **Readmissions (14 days)**   - Low Volume Hospitals - Medium Volume Hospitals - High Volume Hospitals - Very High Volume Hospitals   **Readmissions (42 days)**   - Low Volume Hospitals - Medium Volume Hospitals - High Volume Hospitals - Very High Volume Hospitals   **Dislocations**   - Low Volume Hospitals - Medium Volume Hospitals - High Volume Hospitals - Very High Volume Hospitals   **Reoperations**   - Low Volume Hospitals - Medium Volume Hospitals - High Volume Hospitals - Very High Volume Hospitals   **Infections**   - Low Volume Hospitals - Medium Volume Hospitals - High Volume Hospitals - Very High Volume Hospitals | **Readmissions (14 days)**   - Reference - NS - NS - NS   **Readmissions (42 days)**   - Reference - NS - NS - NS   **Dislocations**   - Reference - NS - *S* - NS   **Reoperations**   - Reference - NS - NS - NS   **Infections**   - Reference - NS - NS - NS |  | **Readmissions (14 days) Adjusted OR**   - Reference - 0.9 (0.8–1.1) - (0.9–1.2) - 1.2 (1.0–1.4)   **Readmissions (42 days) Adjusted OR**   - Reference - 0.9 (0.8–1.0) - 0.9 (0.8–1.1) - 1.1 (1.0–1.2)   **Dislocations Adjusted OR**   - Reference - 0.9 (0.7–1.1) - *0.7 (0.6–0.9)* - 1.1 (0.9–1.4)   **Reoperations**   - Reference - 1.1 (0.8–1.3) - 0.9 (0.8–1.2) - 0.9 (0.7–1.1)   **Infections**   - Reference - (0.6–1.6) - 0.9 (0.6–1.4) - 0.8 (0.5–1.4) |
| **Manley, et al.^(^**[**^25^**](#_ENREF_25)**^)^** | 1-10 | 11-25 | 26-50 | 51-100 | >100 | **Revision Rate 6 Months**   - Very Low Volume Hospitals - Low Volume Hospitals - Medium Volume Hospitals - High Volume Hospitals - Very High Volume Hospitals   **Revision Rate 2 Years**   - Very Low Volume Hospitals - Low Volume Hospitals - Medium Volume Hospitals - High Volume Hospitals - Very High Volume Hospitals   **Revision Rate 5 Years**   - Very Low Volume Hospitals - Low Volume Hospitals - Medium Volume Hospitals - High Volume Hospitals - Very High Volume Hospitals   **Revision Rate 8 Years**   - Very Low Volume Hospitals - Low Volume Hospitals - Medium Volume Hospitals - High Volume Hospitals - Very High Volume Hospitals | **Revision Rate 6 Months**   - NS (P >0.05) - NS (P >0.05) - NS (P >0.05) - NS (P >0.05) - Reference   **Revision Rate 2 Years**   - NS (P >0.05) - NS (P >0.05) - NS (P >0.05) - NS (P >0.05) - Reference   **Revision Rate 5 Years**   - NS (P >0.05) - NS (P >0.05) - NS (P >0.05) - NS (P >0.05) - Reference   **Revision Rate 8 Years**   - NS (P >0.05) - NS (P >0.05) - NS (P >0.05) - NS (P >0.05) - Reference |  | **Revision Rate 6 Months Adjusted HR**   - NA - 0.96 (0.53, 1.74) - 0.88 (0.60, 1.29) - 1.06 (0.77, 1.45) - Reference   **Revision Rate 2 Years Adjusted HR**   - NA - 0.94 (0.62, 1.42) - 0.81 (0.62, 1.06) - 1.02 (0.83, 1.27) - Reference   **Revision Rate 5 Years Adjusted HR**   - NA - 1.03 (0.73, 1.46) - 0.86 (0.69, 1.08) - 1.04 (0.86, 1.24) - Reference   **Revision Rate 8 Years Adjusted HR**   - NA - 0.96 (0.68, 1.35) - 0.90 (0.72, 1.11) - 1.05 (0.88, 1.25) - Reference |
| **Martineau, et al.^(^**[**^26^**](#_ENREF_26)**^)^** |  | Low Vol. |  | High Vol. |  | **Total Cost**   - Low Volume Hospitals - High Volume Hospitals   **Direct Cost**   - Low Volume Hospitals - High Volume Hospitals   **Overhead Cost**   - Low Volume Hospitals - High Volume Hospitals   **Length of Stay**   - Low Volume Hospitals - High Volume Hospitals   **Postoperative Mortality**   - Low Volume Hospitals - High Volume Hospitals | **Total Cost**   - *S (P < 0.0001)*   **Direct Cost**   - *S (P < 0.0001)*   **Overhead Cost**   - *S (P < 0.0001)*   **Length of Stay**   - NS (P >0.05)   **Postoperative Mortality**   - NS (P >0.05) |  | **Total Cost x̅ ± SEM; Median, IQR**   - *7385 ± 138; 6634, 5295–8631* - *4403 ± 117; 3952, 3571–4621*   **Direct Cost x̅ ± SEM; Median, IQR**   - 4952 ± 91; 4646, 3560–5704 - 3023 ± 83; 2685, 2437–3166   **Overhead Cost x̅ ± SEM; Median, IQR**   - 2432 ± 49; 2029, 1641–2890 - 1380 ± 35; 1259, 1132–1461   **Length of Stay x̅ ± SEM; Median, IQR**   - 7.4 ± 9.6; 6.0, 5.0–8.0 - 6.3 ± 3.6; 6.0, 5.0–7.0   **Postoperative Mortality x̅ ± SEM; Median, IQR**   - 0 - 0 |
| **Meyer, et al.^(^**[**^27^**](#_ENREF_27)**^)^** |  | < 50 | 50-100 | >100 |  | **Surgical Site Infections**   - Low Volume Hospitals - Medium Volume Hospitals - High Volume Hospitals | - NS >0.05 - Reference - *S <0.05* | **Incidence /100**   - 1.11 - 1.59 - *0.84* | **Surgical Site Infections OR**   - 0.8110 - Reference - *0.6159* |
| **Mitsuyasu, et al.^(^**[**^28^**](#_ENREF_28)**^)^** |  | < 45 |  | 45-151 |  | **Total Charge**   - Low Volume Hospitals - High Volume Hospitals   **LOS**   - Low Volume Hospitals - High Volume Hospitals   **Preoperative LOS**   - Low Volume Hospitals - High Volume Hospitals   **Postoperative LOS**   - Low Volume Hospitals - High Volume Hospitals | **Mean Total Charge**   - S (P <0.01) - Reference   **Mean LOS**   - S (P <0.01) - Reference   **Preoperative LOS**   - NS (P = 0.92) - Reference   **Postoperative LOS**   - S (P <0.01) - Reference | ¥ (**x̅ ± SD)**   - 2632359.52 ± 1202602.06 - 2347029.11 ± 840339.15   **Days** (**x̅ ± SD)**   - 70.61 ± 40.34 - 50.78 ± 30.34   **Days** (**x̅ ± SD)**   - 11.87 ± 20.59 - 12.02 ± 10.78   **Days** (**x̅ ± SD)**   - 58.73 ±32.31 - 38.76 ± 23.47 |  |
| **Muilwijk, et al.^(^**[**^29^**](#_ENREF_29)**^)^** |  | Low (Tertile) | Medium (Tertile) | High (Tertile) |  | **Surgical Site Infections**   - Low Volume Hospitals - Medium Volume Hospitals - High Volume Hospitals | - Reference - NS (P > 0.001) - NS (P > 0.001) | **Incidence (%)**   - 106/2614 (4.1) - 124/5254 (2.4) - 228/8038 (2.8) | **Surgical Site Infections Adjusted OR**   - Reference - 0.54 (0.27-1.09) - 0.80 (0.42-1.55) |
| **Namba, et al.^(^**[**^30^**](#_ENREF_30)**^)^** |  | < 100 | 100-199 | >200 |  | **Surgical Site Infections**   - Low Volume Hospitals - Medium Volume Hospitals - High Volume Hospitals | - NS =0.554 - NS =0.631 - Reference | **No. of SSI (%)**   - 42 (27.1) - 55 (35.5) - 58 (37.4) | **Surgical Site Infections HR**   - 0.89 (0.60 - 1.32) - 0.91 (0.63 - 1.32) - Reference |
| **Ong, et al.^(^**[**^31^**](#_ENREF_31)**^)^** | 1-10 | 11-25 | 26-50 | 51-100 | >100 | **Duration of Surgery**   - Very Low Volume Hospitals - Low Volume Hospitals - Medium Volume Hospitals - High Volume Hospitals - Very High Volume Hospitals | *S (P<0.0001)* | **Minutes**   - 187.5 - 175 - 163 - 155 - 148.5 |  |
| **Pablo, et al. (2004)^(^**[**^32^**](#_ENREF_32)**^)^** |  | < 40 |  | >40 |  | **Discharge to Rehab. Facility**   - Low Volume Hospitals - High Volume Hospitals | - Reference - NS (P>0.05) |  | **Discharge to Rehabilitation Facility RR**   - Reference - 0.97 (0.78–1.19) |
| **Pablo, et Al (2006)^(^**[**^33^**](#_ENREF_33)**^)^** |  | <16 | 16-49 | >49 |  | **Radiological Follow Up**   - Low Volume Hospitals - Medium Volume Hospitals - High Volume Hospitals | - NS (P>0.05) - NS (P>0.05) - Reference | **Percentage**   - 84% - 89% - 82% | **Radiological Follow Up OR**   - 1.13 (0.79, 1.63) - 1.52 (1.06, 2.19) - Reference |
| **Pamilo, et al. ^(^**[**^34^**](#_ENREF_34)**^)^** |  | 1-199 | 200-499 | 500-899 | >900 | **Readmission 14 days**   - Low Volume Hospitals - Medium Volume Hospitals - High Volume Hospitals - Very High Volume Hospitals   **Readmission 42 days**   - Low Volume Hospitals - Medium Volume Hospitals - High Volume Hospitals - Very High Volume Hospitals   **Reoperations**   - Low Volume Hospitals - Medium Volume Hospitals - High Volume Hospitals - Very High Volume Hospitals | **Readmission 14 days**   - NS (P >0.01) - NS (P>0.01) - NS (P>0.01) - Reference   **Readmission 42 days**   - NS (P>0.01) - NS (P>0.01) - NS (P>0.01) - Reference   **Reoperations**   - NS (P>0.01) - NS (P>0.01) - NS (P>0.01) - Reference |  | **Readmission 14 days Adjusted OR**   - 1.06 (0.96–1.17) - 1.02 (0.93–1.09) - 0.87 (0.79–0.96) - Reference   **Readmission 42 days Adjusted OR**   - 1.14 (1.05–1.23) - 1.01 (0.95–1.09) - 0.92 (0.86–0.99) - Reference   **Reoperations Adjusted OR**   - 1.07 (0.92–1.23) - 1.11 (0.98–1.26) - 0.88 (0.76–1.01) - Reference |
| **Paterson, et al.^(^**[**^35^**](#_ENREF_35)**^)^** |  | 10-110 | 111-150 | 151-225 | >225 | **Mean LOS**   - Low Volume Hospitals - Medium Volume Hospitals - High Volume Hospitals - Very High Volume Hospitals   **Mean LUIC**   - Low Volume Hospitals - Medium Volume Hospitals - High Volume Hospitals - Very High Volume Hospitals   **Death within 90 days**   - Low Volume Hospitals - Medium Volume Hospitals - High Volume Hospitals - Very High Volume Hospitals   **Readmission (Amputation, Fusion or Excision within 1 year)**   - Low Volume Hospitals - Medium Volume Hospitals - High Volume Hospitals - Very High Volume Hospitals   **Readmission (For revision THA)**   - Low Volume Hospitals - Medium Volume Hospitals - High Volume Hospitals - Very High Volume Hospitals   **Acute Hospital Length of Stay**   - Low Volume Hospitals - Medium Volume Hospitals - High Volume Hospitals - Very High Volume Hospitals | **Mean LOS**  S (P<0.01)  **Mean LUIC**  S (P<0.01)  **Death 90D**   - Reference - NS (P>0.05) - NS (P>0.05) - NS (P>0.05)   **Readmission (Amputation, Fusion or Excision within 1Y)**   - Reference - NS (P >0.05) - *S (P <0.05)* - NS (P>0.05)   **Readmission (Revision THA)**   - Reference - NS (P>0.05) - NS (P>0.05) - NS (P>0.05)   **Acute Hospital Length of Stay**   - Reference - NS (P>0.05) - NS (P>0.05) - NS (P>0.05) | **Mean LOS**   - 8.5/1000 - 7.7/1000 - 6.7/1000 - 4.4/1000   **Mean LUIC**   - 11.4/1000 - 11.0/1000 - 10.7/1000 - 7.5/1000   **Death 90D**   - 6.22/1000 - 7.04/1000 - 5.20/1000 - 5.44/1000   **Readmission (A, F, E 1Y)**   - 2.26/1000 - 1.85/1000 - 1.25/1000 - 4.81/1000   **Readmission (Rev. THA)**   - 17.91/1000 - 15.55/1000 - 16.23/1000 - 17.36/1000 | **Death within 90 days Adjusted OR**   - Reference - 1.19 (0.76–1.87) - 0.90 (0.55–1.49) - 1.22 (0.53–2.78)   **Readmission (Amputation, Fusion or Excision within 1 year) Adjusted OR**   - Reference - 0.76 (0.33–1.73) - *0.40 (0.18–0.88)* - 0.76 (0.24–2.40)   **Readmission (For revision THA) Adjusted OR**   - Reference - 0.92 (0.60–1.41) - 0.94 (0.67–1.33) - 1.34 (0.71–2.52)   **Acute Hospital Length of Stay Adjusted OR**   - Reference - 0.97 (0.85–1.11) - 0.93 (0.74–1.17) - 0.79 (0.61–1.00 |
| **Paxton, et al. ^(^**[**^36^**](#_ENREF_36)**^)^** |  | <100 | 100-199 | >199 |  | **30-day Readmission**   - Low Volume Hospitals - Medium Volume Hospitals - High Volume Hospitals | - *S (P = 0.004)* - *S (P = 0.016)* - Reference | **N (%)**   - *78 (4.3)* - *289 (3.7)* - 69 (2.9) | **Adjusted OR**   - *1.81 (1.20–2.72)* - *1.41 (1.07–1.85)* - Reference |
| **Ramkumar, et al. ^(^**[**^37^**](#_ENREF_37)**^)^** | - | 0-120 | 121-357 | ≥ 358 | - | **Length of Stay**   - Low Volume Hospitals - Medium Volume Hospitals - High Volume Hospitals | - *S (P <0.001)* - S (P =0.095) - Reference |  | **OR**   - *17.688 (16.705-18.729)* - 2.712 (2.543-2.894) - Reference |
| **Shi, et al.^(^**[**^38^**](#_ENREF_38)**^)^** |  | 1-99 |  | >99 |  | **Mean Hospital Charge ($)**   - Low Volume Hospitals - High Volume Hospitals | S <0.001 | **Mean ($)**   - 4,816.2 - *3,285.8*   **HVH vs. LVH x̅ (SEM)**  −252.03 (18.69) | **Mean Hospital Charge Adjusted OR**   - Reference - *0.64 (0.48–0.81)* |
| **Singh, et al.^(^**[**^39^**](#_ENREF_39)**^)^** |  | <26 | 26-100 | 100-200 | >200 | **30-day Mortality**   - Low Volume Hospitals - Medium Volume Hospitals - High Volume Hospitals - Very High Volume Hospitals   **1-Year Mortality**   - Low Volume Hospitals - Medium Volume Hospitals - High Volume Hospitals - Very High Volume Hospitals   **Overall Complications**   - Low Volume Hospitals - Medium Volume Hospitals - High Volume Hospitals - Very High Volume Hospitals   **Patients >65 (30-day mortality)**   - Low Volume Hospitals - Medium Volume Hospitals - High Volume Hospitals - Very High Volume Hospitals   **Patients > 65 (1-year mortality)**   - Low Volume Hospitals - Medium Volume Hospitals - High Volume Hospitals - Very High Volume Hospitals   **Patients >65 (Overall Comp.)**   - Low Volume Hospitals - Medium Volume Hospitals - High Volume Hospitals - Very High Volume Hospitals | **30-day Mortality**   - NS (P=0.53) - NS - NS - Reference   **1-Year Mortality**   - *S (P<0.01)* - *S (P<0.01)* - NS (P>0.05) - Reference   **Overall Complications**   - NS (P=0.40) - NS - NS - Reference   **Patients >65 (30-day mortality)**   - NS (P=0.32) - NS - NS - Reference   **Patients > 65 (1-year mortality)**   - *S (P<0.01)*   **Patients >65 (Overall Comp.)**   - NS (P=0.16) - NS - NS - NS | **30D Mortality**   - 6/814 - 29/4,163 - 9/2,246 - 9/2,964   **1Y Mortality**   - 32/814 - 147/4,163 - 50/2,246 - 25/2,964   **Overall Comp.**   - 25/814 - 129/4,163 - 57/2,246 - 67/2,964   **>65 30D Mort.**   - 5/566 - 27/2,757 - 8/1,397 - 7/1,536   **>65 1Y Mort.**   - *26/566* - 127/2,757 - 45/1,397 - 42/1,536   **>65 Ov. Comp.**   - 16/566 - 94/2,757 - 39/1,397 - 47/1,536 | **30-day Mortality**   - 0.9 (0.2–4.2) - 1.6 (0.6–4.1) - 1.3 (0.4–4.5) - Reference   **1-Year Mortality**   - 2.1 (1.2–3.6) - 2.0 (1.4–2.9) - (0.7–1.5) - Reference   **Overall Complications**   - 1.3 (0.6–2.5) - 1.5 (0.9–2.4) - 1.3 (0.7–2.3) - Reference   **Patients >65 (30-day mortality)**   - 1.0 (0.2–4.6) - 1.9 (0.7–4.9) - 1.1 (0.3–4.5) - Reference   **Patients > 65 (1-year mortality)**   - *2.2 (1.2–4.3)* - 2.2 (1.4–3.4) - 1.0 (0.6–1.5) - Reference   **Patients >65 (Overall Comp.)**   - 1.0 (0.4–2.3) - 1.4 (0.8–2.6) - 1.1 (0.6–2.1) - Reference |
| **Solomon, et al. ^(^**[**^40^**](#_ENREF_40)**^)^** | - | 1-25 | 26-100 | >100 | - | **90-day Adverse Event**   - Low Volume Hospitals - Medium Volume Hospitals - High Volume Hospitals | - Reference - NS (P = 0.4) - *S (P < 0.001)* | **Percentage**   - 3.6% - 2.8% - *1.4%* | **Adjusted OR**   - Reference - 0.84 (0.57–1.25) - *0.44 (0.28–0.70)* |
| **Soohoo, et al. ^(^**[**^41^**](#_ENREF_41)**^)^** | - | Lower 40^th^ Percentile | 40-80^th^  Percentile | Top 20^th^ Percentile | - | **Overall complications**   - Low Volume Hospitals - Medium Volume Hospitals - High Volume Hospitals   **90-day Mortality**   - Low Volume Hospitals - Medium Volume Hospitals - High Volume Hospitals   **90-day Infection**   - Low Volume Hospitals - Medium Volume Hospitals - High Volume Hospitals   **90-day Dislocation**   - Low Volume Hospitals - Medium Volume Hospitals - High Volume Hospitals   **90-day Revision**   - Low Volume Hospitals - Medium Volume Hospitals - High Volume Hospitals   **90-day TE**   - Low Volume Hospitals - Medium Volume Hospitals - High Volume Hospitals | **Overall Complication**   - *S (P <0.001)* - *S (P <0.001)* - Reference   **90-day Mortality**   - *S (P <0.001)* - *S (P <0.001)* - Reference   **90-day Infection**   - *S (P <0.001)* - *S (P <0.001)* - Reference   **90-day Dislocation**   - *S (P <0.001)* - *S (P<0.001)* - Reference   **90-day Revision**   - *S (P <0.001)* - NS (P = 0.05) - Reference   **90-day Thromboembolism**   - *S (P <0.001)* - *S (P <0.001)* - Reference | - | **Overall Complication OR**   - *2.00 (1.82–2.20)* - *1.33 (1.22–1.45)* - Reference   **90-day Mortality OR**   - *1.82 (1.44–2.30)* - *1.45 (1.17–1.79)* - Reference   **90-day Infection OR**   - *2.35 (1.87–2.94)* - *1.48 (1.20–1.83)* - Reference   **90-day Dislocation OR**   - *2.43 (2.08–2.84)* - *1.40 (1.21–1.62)* - Reference   **90-day Revision OR**   - *1.78 (1.42–2.22)* - 1.22 (1.00–1.49) - Reference   **90-day Thromboembolism OR**   - *1.78 (1.42–2.22)* - *1.22 (1.00–1.49)* - Reference |
| **Styron, et al. ^(^**[**^42^**](#_ENREF_42)**^)^** |  | 1-64 | 65-123 | 124-224 | ≥225 | **Mean Length of Stay**   - Low Volume Hospitals - Medium Volume Hospitals - High Volume Hospitals - Very High Volume Hospitals | - *S (P < 0.0001)* | **Diff. LOS x̅**   - 1.146 - 1.055 - 1.030 - Reference | **Mean Transformed LOS (95% CI)**   - 4.17 (2.69, 5.64) - 3.91 (2.47, 5.34) - 3.86 (2.47, 5.24) - 3.72 (2.27, 5.16) |

| **Table 4: Hospital Volume Thresholds and Outcomes in Studies of Revision Total Hip Arthroplasty (rTHA)** | | | | | | | | | |
| --- | --- | --- | --- | --- | --- | --- | --- | --- | --- |
| **Study** | **Hospital Volume Thresholds for rTHA** | | | | | **Outcomes** | **Significance** | **Outcome Results** | **OR/ RR/HR/Rate/Others [95% CI]** |
|  | **Very Low** | **Low** | **Medium** | **High** | **Very High** |  |  |  |  |
| **Katz, et al. (2001) ^(^**[**^14^**](#_ENREF_14)**^)^** | 1-10 | 11-25 | 26-50 | 51-100 | >100 | **90-day Dislocation**   - Very Low Volume Hospitals - Low Volume Hospitals - Medium Volume Hospitals - High Volume Hospitals - Very High Volume Hospitals   **90-day Deep Infection**   - Very Low Volume Hospitals - Low Volume Hospitals - Medium Volume Hospitals - High Volume Hospitals - Very High Volume Hospitals   **90-day Pulmonary Embolism**   - Very Low Volume Hospitals - Low Volume Hospitals - Medium Volume Hospitals - High Volume Hospitals - Very High Volume Hospitals   **90- day Mortality**   - Very Low Volume Hospitals - Low Volume Hospitals - Medium Volume Hospitals - High Volume Hospitals - Very High Volume Hospitals | **90-day Dislocation**   - Reference - NS (P>0.01) - NS (P>0.01) - NS (P>0.01) - NS (P>0.01)   **90-day Deep Infection**   - Reference - NS (P>0.01) - NS (P>0.01) - NS (P>0.01) - NS (P>0.01)   **90-day Pulmonary Embolism**   - Reference - NS (P>0.01) - NS (P>0.01) - NS (P>0.01) - NS (P>0.01)   **90- day Mortality**   - Reference - NS (P>0.01) - NS (P>0.01) - NS (P>0.01) - NS (P>0.01) | **Percentage**   - 9.8% - 8.6% - 8.4% - 7.0% - 4.2%   **Percentage**   - 0.9% - 1.1% - 1.0% - 0.9% 0.5%   **Percentage**   - 0.7% - 1.1% - 0.7% - 0.5% - 0.7%   **Percentage**   - 3.5% - 2.6% - 2.1% - 1.5% - 1.5% | **90-day Dislocation Adjusted OR**   - Reference - 0.90 (0.75, 1.08) - 0.90 (0.75, 1.09) - 0.75 (0.56, 1.02) - 0.45 (0.30, 0.66)   **90-day Deep Infection Adjusted OR**   - Reference - 1.31 (0.78, 2.21) - 1.39 (0.84, 2.31) - 1.36 (0.64, 2.92) - 0.78 (0.29, 2.10)   **90-day Pulmonary Embolism Adjusted OR**   - Reference - 1.63 (0.94, 2.81) - 1.01 (0.54, 1.90) - 0.67 (0.29, 1.57) - 0.91 (0.40, 2.06)   **90- day Mortality Adjusted OR**   - Reference - 0.85 (0.62, 1.15) - 0.74 (0.54, 1.00) - 0.67 (0.40, 1.11) - 0.85 (0.43, 1.67) |
| **Katz, et al. (2003) ^(^**[**^15^**](#_ENREF_15)**^)^** |  | 1-30 | 31-100 | >100 |  | **Harris Hip score in lowest 10%**   - Low Volume Hospitals - Medium Volume Hospitals - High Volume Hospitals   **Satisfaction Score < 50**   - Low Volume Hospitals - Medium Volume Hospitals - High Volume Hospitals | **Harris Hip Score Lowest (10%)**   - NS (P>0.05) - NS (P>0.05) - Reference   **Satisfaction Score < 50**   - NS >0.05 - NS >0.05 - Reference | **Percentage**   - 13.6 - 9.6 - 7.6   **Percentage**   - 29.7 - 27.4 - 25.2 | **Harris Hip Score in Lowest 10% Adjusted OR**   - 0.90 (0.40–1.99) - 0.94 (0.45–1.95) - Reference   **Satisfaction Score < 50 Adjusted OR**   - 0.81 (0.44–1.48) - 0.85 (0.54–1.33) - Reference |
| **Muilwijk, et al. ^(^**[**^29^**](#_ENREF_29)**^)^** |  | Low  (Tertile) | Medium (Tertile) | High (Tertile) |  | **Surgical Site Infections**   - Low Volume Hospitals - Medium Volume Hospitals - High Volume Hospitals | - Reference - NS (P > 0.001) - NS (P > 0.001) | **Incidence (%)**   - 8/139 (5.8) - 24/337 (7.1) - 43/570 (7.5) | **Surgical Site Infections Adjusted OR**   - Reference - 1.47 (0.35-6.13) - 1.23 (0.30-5.09) |
| **Pablo, et al. (2004) ^(^**[**^32^**](#_ENREF_32)**^)^** |  | <40 |  | >40 |  | **Discharge to Rehab. Facility**   - Low Volume Hospitals - High Volume Hospitals | - Reference - NS (P>0.05) |  | **Discharge to Rehabilitation Facility RR**   - Reference - 0.95 (0.73–1.22) |

**References**

1. Adelani MA, Keller MR, Barrack RL, Olsen MA. The impact of hospital volume on racial differences in complications, readmissions, and emergency department visits following total joint arthroplasty. The Journal of arthroplasty. 2018;33(2):309-15. e20.

2. Boas R, Ensor K, Qian E, Hutzler L, Slover J, Bosco J. The relationship of hospital charges and volume to surgical site infection after total hip replacement. American journal of medical quality. 2015;30(3):283-8.

3. Cécile Camberlin M. Provider volume and short term complications after elective total hip replacement: an analysis of Belgian administrative data. Acta Orthopædica Belgica. 2011;77:311-9.

4. Le Cossec C, Colas S, Zureik M. Relative impact of hospital and surgeon procedure volumes on primary total hip arthroplasty revision: a nationwide cohort study in France. Arthroplasty today. 2017;3(3):176-82.

5. Doro C, Dimick J, Wainess R, Upchurch G, Urquhart A. Hospital volume and inpatient mortality outcomes of total hip arthroplasty in the United States. The Journal of arthroplasty. 2006;21(6):10-6.

6. Dy CJ, Bozic KJ, Pan TJ, Wright TM, Padgett DE, Lyman S. Risk factors for early revision after total hip arthroplasty. Arthritis care & research. 2014;66(6):907-15.

7. Espehaug B, Havelin LI, Engesaeter LB, Vollset SE. The effect of hospital-type and operating volume on the survival of hip replacements: a review of 39,505 primary total hip replacements reported to the Norwegian Arthroplasty Register, 1988-1996. Acta orthopaedica Scandinavica. 1999;70(1):12-8.

8. Glassou E, Hansen T, Mäkelä K, Havelin LI, Furnes O, Badawy M, et al. Association between hospital procedure volume and risk of revision after total hip arthroplasty: a population-based study within the Nordic Arthroplasty Register Association database. Osteoarthritis and cartilage. 2016;24(3):419-26.

9. Helkamaa T, Hirvensalo E, Huhtala H, Remes V. Patient injuries in primary total hip replacement: Nationwide analysis in Finland. Acta orthopaedica. 2016;87(3):209-17.

10. Huang C-S, Cheu Y-D, Ying J, Wei M-H. Association between provider volume and comorbidity on hospital utilization and outcomes of total hip arthroplasty among National Health Insurance enrollees. Journal of the Formosan Medical Association. 2011;110(6):401-9.

11. Hughes RG, Garnick DW, Luft HS, McPhee SJ, Hunt SS. Hospital volume and patient outcomes. The case of hip fracture patients. Med Care. 1988;26(11):1057-67. Epub 1988/11/01.

12. Järvelin J, Häkkinen U, Rosenqvist G, Remes V. Factors predisposing to claims and compensations for patient injuries following total hip and knee arthroplasty. Acta orthopaedica. 2012;83(2):190-6.

13. Kaneko T, Hirakawa K, Fushimi K. Relationship between peri-operative outcomes and hospital surgical volume of total hip arthroplasty in Japan. Health Policy. 2014;117(1):48-53.

14. Katz JN, Losina E, Barrett J, Phillips CB, Mahomed NN, Lew RA, et al. Association between hospital and surgeon procedure volume and outcomes of total hip replacement in the United States Medicare population. Jbjs. 2001;83(11):1622-9.

15. Katz JN, Phillips CB, Baron JA, Fossel AH, Mahomed NN, Barrett J, et al. Association of hospital and surgeon volume of total hip replacement with functional status and satisfaction three years following surgery. Arthritis and rheumatism. 2003;48(2):560-8. Epub 2003/02/07.

16. Katz JN, Wright EA, Wright J, Malchau H, Mahomed NN, Stedman M, et al. Twelve-year risk of revision after primary total hip replacement in the US Medicare population. The Journal of bone and joint surgery American volume. 2012;94(20):1825.

17. Khatod M, Cafri G, Namba RS, Inacio MC, Paxton EW. Risk factors for total hip arthroplasty aseptic revision. The Journal of arthroplasty. 2014;29(7):1412-7.

18. Khuri SF, Daley J, Henderson W, Hur K, Hossain M, Soybel D, et al. Relation of surgical volume to outcome in eight common operations: results from the VA National Surgical Quality Improvement Program. Annals of surgery. 1999;230(3):414.

19. Kreder HJ, Deyo RA, Koepsell T, Swiontkowski MF, Kreuter W. Relationship between the volume of total hip replacements performed by providers and the rates of postoperative complications in the state of Washington. Jbjs. 1997;79(4):485-94.

20. Kreder HJ, Williams JI, Jaglal S, Hu R, Axcell T, Stephen D. Are complication rates for elective primary total hip arthroplasty in Ontario related to surgeon and hospital volumes? A preliminary investigation. Canadian journal of surgery. 1998;41(6):431.

21. de Vries LM, Sturkenboom MC, Verhaar JA, Kingma JH, Stricker BH. Complications after hip arthroplasty and the association with hospital procedure volume: a nationwide retrospective cohort study on 50,080 total hip replacements with a follow-up of 3 months after surgery. Acta orthopaedica. 2011;82(5):545-52.

22. Losina E, Barrett J, Mahomed NN, Baron JA, Katz JN. Early failures of total hip replacement: effect of surgeon volume. Arthritis & Rheumatism: Official Journal of the American College of Rheumatology. 2004;50(4):1338-43.

23. Maceroli MA, Nikkel LE, Mahmood B, Elfar JC. Operative mortality after arthroplasty for femoral neck fracture and hospital volume. Geriatric orthopaedic surgery & rehabilitation. 2015;6(4):239-45.

24. Mäkelä KT, Häkkinen U, Peltola M, Linna M, Kröger H, Remes V. The effect of hospital volume on length of stay, re-admissions, and complications of total hip arthroplasty: a population-based register analysis of 72 hospitals and 30,266 replacements. Acta orthopaedica. 2011;82(1):20-6.

25. Manley M, Ong K, Lau E, Kurtz SM. Effect of volume on total hip arthroplasty revision rates in the United States Medicare population. Jbjs. 2008;90(11):2446-51.

26. Martineau P, Filion KB, Huk OL, Zukor DJ, Eisenberg MJ, Antoniou J. Primary hip arthroplasty costs are greater in low-volume than in high-volume Canadian hospitals. Clinical Orthopaedics and Related Research®. 2005;437:152-6.

27. Meyer E, Weitzel-Kage D, Sohr D, Gastmeier P. Impact of department volume on surgical site infections following arthroscopy, knee replacement or hip replacement. BMJ Qual Saf. 2011;20(12):1069-74.

28. Mitsuyasu S, Hagihara A, Horiguchi H, Nobutomo K. Relationship between total arthroplasty case volume and patient outcome in an acute care payment system in Japan. The Journal of arthroplasty. 2006;21(5):656-63.

29. Muilwijk J, van den Hof S, Wille JC. Associations between surgical site infection risk and hospital operation volume and surgeon operation volume among hospitals in the Dutch nosocomial infection surveillance network. Infection Control & Hospital Epidemiology. 2007;28(5):557-63.

30. Namba R, Inacio M, Paxton E. Risk factors associated with surgical site infection in 30 491 primary total hip replacements. The Journal of bone and joint surgery British volume. 2012;94(10):1330-8.

31. Ong K, Lau E, Manley M, Kurtz SM. Patient, hospital, and procedure characteristics influencing total hip and knee arthroplasty procedure duration. The Journal of arthroplasty. 2009;24(6):925-31.

32. Pablo Pd, Losina E, Phillips CB, Fossel AH, Mahomed N, Lingard EA, et al. Determinants of discharge destination following elective total hip replacement. Arthritis care & research. 2004;51(6):1009-17.

33. de Pablo P, Losina E, Mahomed N, Wright J, Fossel AH, Barrett JA, et al. Extent of followup care after elective total hip replacement. The Journal of Rheumatology. 2006;33(6):1159.

34. Pamilo KJ, Peltola M, Mäkelä K, Häkkinen U, Paloneva J, Remes V. Is hospital volume associated with length of stay, re-admissions and reoperations for total hip replacement? A population-based register analysis of 78 hospitals and 54,505 replacements. Archives of orthopaedic and trauma surgery. 2013;133(12):1747-55.

35. Paterson JM, Williams JI, Kreder HJ, Mahomed NN, Gunraj N, Wang X, et al. Provider volumes and early outcomes of primary total joint replacement in Ontario. Canadian journal of surgery. 2010;53(3):175.

36. Paxton EW, Inacio MC, Singh JA, Love R, Bini SA, Namba RS. Are there modifiable risk factors for hospital readmission after total hip arthroplasty in a US healthcare system? Clinical Orthopaedics and Related Research®. 2015;473(11):3446-55.

37. Ramkumar PN, Navarro SM, Frankel WC, Haeberle HS, Delanois RE, Mont MA. Evidence-based thresholds for the volume and length of stay relationship in total hip arthroplasty: outcomes and economies of scale. The Journal of arthroplasty. 2018;33(7):2031-7.

38. Shi H-Y, Chang J-K, Chiu H-C. Volume associations in total hip arthroplasty: a nationwide Taiwan population-based study. The Journal of arthroplasty. 2013;28(10):1834-8.

39. Singh JA, Kwoh CK, Boudreau RM, Lee GC, Ibrahim SA. Hospital volume and surgical outcomes after elective hip/knee arthroplasty: A risk‐adjusted analysis of a large regional database. Arthritis & Rheumatism. 2011;63(8):2531-9.

40. Solomon DH, Losina E, Baron JA, Fossel AH, Guadagnoli E, Lingard EA, et al. Contribution of hospital characteristics to the volume–outcome relationship: dislocation and infection following total hip replacement surgery. Arthritis & Rheumatism. 2002;46(9):2436-44.

41. SooHoo NF, Farng E, Lieberman JR, Chambers L, Zingmond DS. Factors that predict short-term complication rates after total hip arthroplasty. Clinical Orthopaedics and Related Research®. 2010;468(9):2363-71.

42. Styron JF, Koroukian SM, Klika AK, Barsoum WK. Patient vs provider characteristics impacting hospital lengths of stay after total knee or hip arthroplasty. The Journal of arthroplasty. 2011;26(8):1418-26.e262. Epub 2011/01/28.
